# Sphingosine 1-phosphate protects against radiation-induced ovarian injury in female rats—impact on mitochondrial-related genes

**DOI:** 10.1186/s12958-020-00659-z

**Published:** 2020-10-12

**Authors:** Jiahui Zhao, Shuyun Zhang, Liesong Chen, Xiaolong Liu, Haihong Su, Lili Chen, Li Yang, Hong Zhang

**Affiliations:** 1 Department of Reproductive Medicine, The Second Affiliated Hospital of Soochow University, NO.1055 SanXiang Road, Suzhou, 215004 Jiangsu Province China; 2 Department of Reproductive Medicine, Lianyungang Maternal and Child Health Hospital, NO.669 Qindongmen Road, Lianyungang, 222001 Jiangsu Province China

**Keywords:** X-ray, Sphingosine 1-phosphate, Ovary, Mitochondria, mRNA

## Abstract

The toxic effects of ionizing radiation on the gonads have been widely recognized. Sphingosine 1-phosphate (S1P) has a protective effect on ovarian injury, and although it is known that mitochondria are involved in this process, the specific mechanism is not fully understood. The present study analysed the changes in the serum AMH and ovarian histology in Sprague-Dawley female rats exposed to X-ray radiation only or co-administered with S1P. The mRNA expression profile of ovarian tissue was further analysed via next-generation sequencing and bioinformatics approaches to screen out candidate mitochondria-related genes. Finally, differentially expressed target genes were verified by real-time PCR. The results showed that ionizing radiation could reduce the serum AMH level, destroy ovarian structure and decrease the number of follicles in rats, while S1P administration significantly attenuated the impairment of ovarian function. Gene ontology (GO) and KEGG pathway analysis revealed that a variety of genes related to mitochondrial function were differentially expressed, and the protective effect of S1P on mitochondria was more obvious in the acute phase 24 h after radiation. The differentially expressed mitochondrial function-related genes associated with the protective effect of S1P were UQCRH, MICU2 and GPX4, which were subsequently verified by RT-PCR. Therefore, ionizing radiation has a significant effect on ovarian function, and S1P has a protective effect on radiation-induced ovarian injury, in which mitochondria may play an important role. This study sheds new light on the mechanism of radiation-induced ovarian injury and helps develop a novel potential strategy to control it.

## Introduction

Ionizing radiation refers to the radiation that carries enough energy to make the electrons in atoms or molecules become unbound from their orbit and thus produce an ionization effect. Ionizing radiation is not only widespread in nature but also one of the principal methods of clinical examination and treatment in medicine. For example, radiotherapy is a widely accepted form of treatment that measures for malignant tumours. It is extensively used in the preoperative management of tumours with risk factors and the treatment of locally advanced tumours, and is one of the salvage treatment measures for recurrent cervical cancer [1, 2]. However, the toxic effect of radiation on the gonads should not be neglected. In females, ovaries are one of the organs that are highly sensitive to ionizing radiation. Radiation has a profound effect on ovarian function; this effect is characterized by follicular atrophy and reduced follicular storage. This can accelerate the natural decline in the number of follicles, leading to impaired ovarian hormone secretion, uterine dysfunction due to insufficient oestrogen exposure, early menopause and infertility. Injury caused by radiation can not only significantly reduce female fertility but also have an impact on the health of future generations, and these adverse consequences are irreversible [3, 4]. Although there are currently several options for fertility preservation before radiation exposure (such as controlled ovarian stimulation, embryos, oocytes and ovarian tissue cryopreservation), it is still important to explore the specific mechanism of radiation-induced ovarian injury and how to protect ovarian function as much as possible during radiation exposure. Mitochondria are the “energy factory” of cells that have complex functions beyond providing energy. Mitochondria have been extensively studied in many fields such as reproduction, inheritance, development and ageing. Mitochondria also play an important role in radiation-induced cellular responses and may participate in the process of radiation damage, but the specific role and mechanism still need to be fully elucidated [5].

Sphingosine 1-phosphate (S1P) is a sphingomyelin metabolite, a vital signalling molecule that participates in a variety of important cell signalling pathways and physiological processes. S1P exhibits various biological activities such as promoting cell growth, inhibiting cell apoptosis and regulating angiogenesis by interacting with five G-protein-coupled receptors (GPCR) [6]. S1P plays an important role in ovarian physiology, participating as an essential stimulator of follicular development in both the preantral and antral phases, as well as in ovulation and corpus luteum development [7]. The protective effect of S1P on the ovaries has been confirmed in several previous studies [8, 9], and other studies have shown that analogues of S1P can regulate mitochondrial oxidative stress [10]; however, the radioprotective mechanism of S1P and the role of mitochondria in ovarian protection are still unclear.

In this present study, a rat model of radiation-induced ovarian injury was utilized to verify the radioprotective effect of S1P. Furthermore, we intended to investigate the role of mitochondrial-related genes that participate in radiation-induced ovarian damage and the protective effects of S1P. Our findings may provide novel insights into the role of mitochondrial-related genes in the radiation-induced ovarian injury process and the mechanisms underlying the ovarian protective effects of S1P.

## Materials and methods

### Animals and experimental design

Experimental Sprague-Dawley female rats were purchased and raised in the Experimental Animal Center of Soochow University. An ambient temperature of 18–24 °C and a continuous 12-h light/dark cycle were maintained. Each rat was given free access to a standard laboratory diet and water. Eighteen rats aged 6–8 weeks and weighing 198.4 ± 10.6 g were randomly divided into a control group, a radiation group and a radiation+S1P group (six rats in each group). In the radiation+S1P group, the rats were intraperitoneally injected with a S1P solution (0.5 mg/kg, Tocris Bioscience, Minneapolis, MN, USA) once a day starting 72 h before radiation until they were sacrificed [11]**.** Rats in the control group and the radiation group were given intraperitoneal injections of an equal volume of solvent for dissolving S1P (PBS with a final concentration of 4 mg/ml BSA) at the same time until they were sacrificed. Rats in the radiation group and the radiation +S1P group were subjected to 6-MV X-ray radiation to the lower abdominal reproductive system area (total dose 2Gy). Twenty-four hours after radiation, three rats were randomly sacrificed in each group and were respectively denoted as the C1 (control), R1 (radiation) and S1 (radiation +S1P) groups. Seventy-two hours after radiation, the remaining rats were sacrificed and recorded as the C2 (control), R2 (radiation) and S2 (radiation +S1P) groups. Serum was obtained from the left ventricle of each group before execution, and the AMH level was detected by ELISA. The right ovary of each group was used for mRNA sequencing, and the left ovary was used for a histopathological examination. All of the animal experiments were approved by the ethics committee of Soochow University and conducted in accordance with its guidelines.

### Serum AMH level detection

Rats were anaesthetized by an intraperitoneal injection of 7% chloral hydrate (0.5 ml/kg) and subjected to an operation in which the chest and abdominal cavity were opened, 3 ~ 5 ml blood was collected from the left ventricle using a 5 ml sterile syringe, and then the blood was placed in a blank tube. The blood was left to stand and centrifuged at 1000×g for 15 min after full coagulation to obtain the serum, which was stored at − 80 °C, and then the AMH level was detected according to the instructions of the Rat AMH ELISA kit (ImmunoWay biotechnology, Plano, TX, USA).

### Histomorphological evaluation of rat ovaries

The left ovary of each group was fixed with 10% neutral formaldehyde, then dehydrated, embedded in paraffin, and sectioned (thickness 4 μm). One slice at an interval of 10 serial sections from the largest cross-section of the ovary was chosen, and a total of three slices of each ovary were selected for haematoxylin/eosin (HE) staining. The ovarian tissue morphology was observed under a light microscope, and the number of normal follicles was counted. Normal follicles had a complete layer of flattened granulosa cells, oocytes with cytoplasm, and a non-pyknotic nucleus. The criteria for classifying developmental stages of ovarian follicles were as follows: the primordial follicles-oocyte was enveloped by a single layer of flattened granulosa cells; the primary follicle-oocyte was surrounded by a single layer of cubic granulosa cells; the secondary follicle-large oocytes was surrounded by layers of granulosa cells; and the antral follicle was the largest follicle with a multilayer granulosa cell enclosing the follicular cavity. The oocyte was surrounded by granulosa cells on one side of the follicular cavity.

### RNA isolation of rat ovarian tissue

The ovarian tissue used for sequencing was isolated and immediately stored in RNA later (Thermo Fisher Scientific, Waltham, MA, USA), which was extracted from tissue samples using a TRIZOL reagent (Invitrogen life technologies, Carlsbad, CA, USA). After RNA extraction, the RNA concentration of each sample was measured using a NanoDrop ND-1000 instrument (Thermo Fisher Scientific, Waltham, MA, USA). The OD260/OD280 ratio was applied to assess the RNA purity. If the OD260/OD280 ratio ranged from 1.8 to 2.1, the RNA purity was acceptable. RNA integrity and gDNA contamination were checked by denatured agarose gel electrophoresis.

### mRNA library construction and sequencing

High-throughput sequencing was conducted by Cloudseq Biotech (Shanghai, China). An NEBNext rRNA Depletion Kit (New England Biolabs, Ipswich, MA, USA) was used to remove rRNA from the sample. The sequencing library was constructed using the NEBNext® Ultra™ II Directional RNA Library Prep Kit (New England Biolabs, Ipswich, MA, USA). The library was quality controlled and quantified by the BioAnalyzer 2100 system (Agilent Technologies, Santa Clara, CA, USA), and 150 bp double-end sequencing was performed using the Illumina Hiseq instrument. After image recognition and base recognition, the original reads were harvested from the Illumina HiSeq sequencer. Taking Q30 as the quality control standard, Q30 > 80% indicated that the sequencing quality was acceptable. Cutadapt software (v1.9.3) was used to remove joints as well as low quality reads and obtain high quality clean reads. Hisat2 software (v2.0.4) was used to compare clean reads with the rat reference genome (RN6), HTSeq software (v0.9.1) was used to obtain the original count, and edgeR was used to normalize the original count and calculate the differentially expressed genes (mRNA) between the two groups of samples. The threshold for differential screening was set as fold change ≧ 2.0 (log FC≧1.0) and a *p*-value ≦ 0.05. After obtaining the differentially expressed mRNA screened by high-throughput sequencing, bioinformatics analysis including GO enrichment analysis (http://geneontology.org/page/go-enrichment-analysis) and KEGG pathway analysis (http://www.genome.jp/kegg/pathway.html) were further performed to determine the potential metabolic pathways and genes related to mitochondria.

### Validation of differentially expressed mRNAs using real-time quantitative PCR

#### Primers for mRNA

Real-time PCR was performed to verify the differentially expressed mRNAs related to mitochondrial function screened by high-throughput sequencing and further filtered by GO and KEGG pathway analysis. The primers that were used are shown in Table 1. The data were analysed by 2 ^-△△CT^.

**Table 1:** Primers for real time PCR

Gene	Types of primers	Gene sequence
Uqcrh	Forward	TTTGACTTCCTGCATGCACG
	Reverse	CAAGCTGTTCCGATTCCCAG
Micu2	Forward	TGTGCTTTCTGGAGGGCTAA
	Reverse	CTACTCCCTAGGCCACCATG
Gpx4	Forward	TAAGTACAGGGGTTGCGTGT
	Reverse	AGGCCAGGATTCGTAAACCA
ACTB (internal reference)	Forward	AAGTCCCTCACCCTCCCAAAAG
	Reverse	AAGCAATGCTGTCACCTTCCC

*PCR* Polymerase chain reaction

#### RNA extraction and quality control

The procedures were the same as those described in the mRNA expression profile analysis section.

#### cDNA synthesis

The annealing mixture contained 0.8 μg of RNA, 1 μl of 0.5 μg/ul Oligo (dT) 18, 1.6 μl of dNTPs Mix (2.5 mM), and RNase-free water to a total volume of 20 μl. Then, it was incubated in a thermal cycler at 65 °C for 5 min and placed on ice for 2 min. The contents of the tube were collected by brief centrifugation before the following were added to the tube: 5 × First-Strand Buffer, 4 μl, 0.1 M DTT, 1 μl, RNase Ihibitor, 0.3 μl, and SuperScript III RT, 0.2 μl. The pipette was gently sucked and tapped several times to obtain a better mixture. Incubation was done as follows: 60 min at 50 °C and then 15 min at 70 °C. The cDNA synthesis reaction was stored at − 20 °C, or PCR was performed immediately [12].

#### Quantitative real-time PCR

RT-qPCR reactions were conducted with a QuantStudio 5 real-time PCR system (Thermo Fisher Scientific, Waltham, MA USA). The real time PCR mixture contained 2 × Master Mix, 5 μl, PCR specific primer F (10 μM), 0.5 μl, PCR specific primer R (10 μM), 0.5 μl and water to a total volume of 8 μl. After the solution was mixed and centrifuged at 5000 RPM for a short time, the reaction mixtures (8 μl) were added into a 384-well PCR plate, and the cDNA samples (2 μl) were added. The plate was sealed and placed on ice. The PCR was initiated by heating the mixture to 95 °C for 10 min, followed by 40 cycles of 10 s at 95 °C and 60 s at 60 °C. To establish the melting curve, the mixture was heated to 95 °C for 10 s, 60 °C for 60 s, and 95 °C for 15 s sequentially after the amplification reaction was over. The data were calculated by the 2 ^-△△CT^ method [13].

### Statistical analysis

SPSS software version 22.0 (IBM, Chicago, IL, USA) was used for statistical analysis of the data, a chi-square test was used for measurement of the data, and the test level was set as α = 0.05. The measurement data were expressed as mean ± standard deviation (mean ± SD). The data were tested for normality, and one-way ANOVA was used for normal distribution. The non-parametric Kruskal-Wallis test was used for data that did not conform to a normal distribution. *P* < 0.05 was considered statistically significant. Bar graphs were created by GraphPad Prism version 5.0 (GraphPad Software, La Jolla, CA, USA).

## Results

### Serum AMH level

**Fig. 1:**
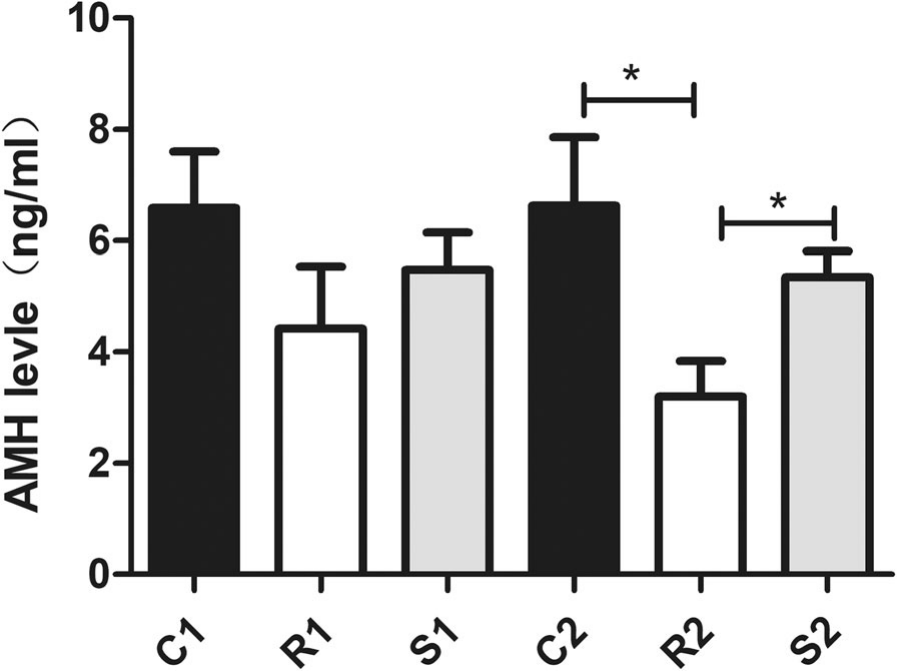
Bar graphics representing the alterations of serum AMH level in all groups. Results were presented as mean ± SEM. *p < 0.05

As shown in Fig. 1, 24 h after radiation, the serum AMH level of the rats in group R1 decreased when compared with the control group, while the serum AMH level of the rats in group S1 increased when compared with group R1, but no statistically significant difference was found (*p* > 0.05). Seventy-two hours after radiation, the serum AMH level of the R2 group was lower than that of the control group, and the serum AMH level of the S2 group was higher than that of the R2 group, with statistically significant differences (*p* < 0.05). Although the serum AMH level of the rats in the S2 group was lower than that in the C2 group, the difference was not statistically significant (*p* > 0.05). The administration of S1P prevented a decrease in AMH levels when compared to the radiation groups.

### Changes in ovarian histology and follicle counts

**Fig. 2:**
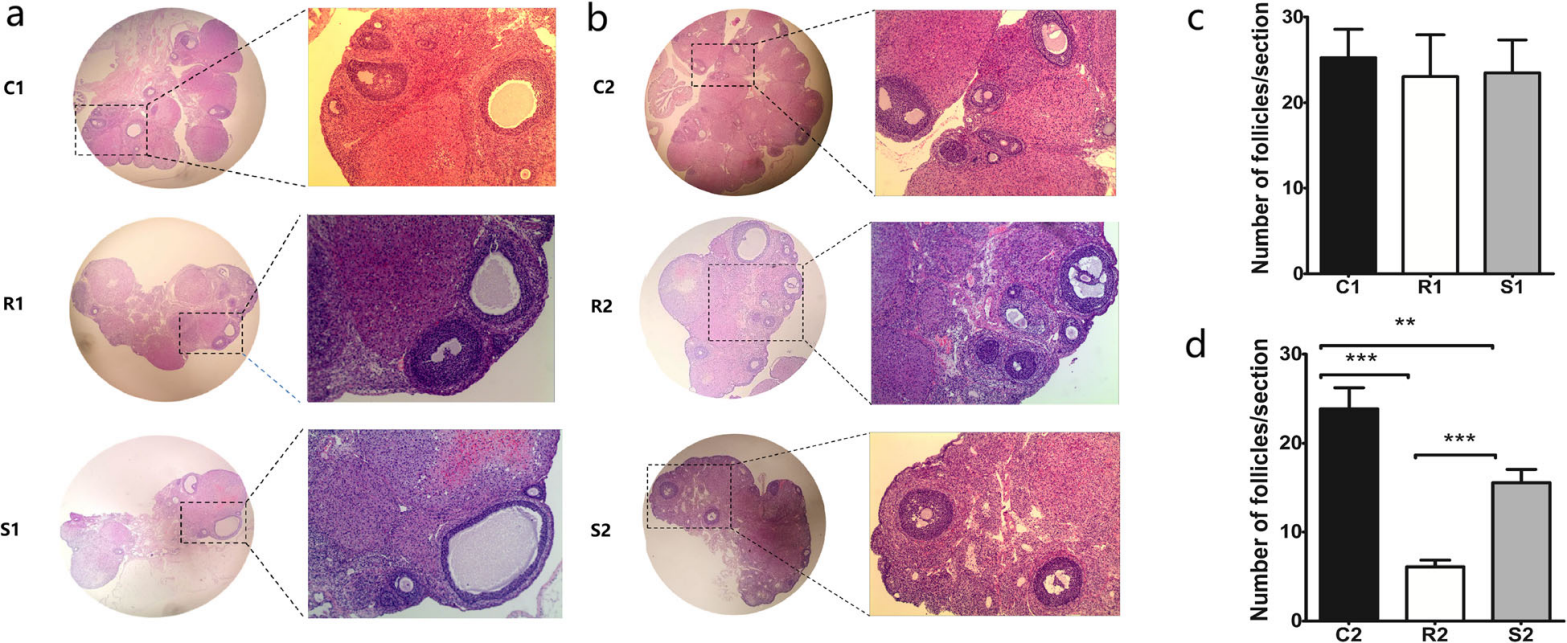
Histopathological examination of ovaries with H&E staining. **a**: 24 h after irradiation, the ovarian tissue in each group was normal, with no obvious structural damage. **b**: 72 h after irradiation, the morphology of the ovaries was obviously damaged by irradiation with hemorrhage in the cortex and ovarian interstitial hyperplasia, and the ovarian structure in group S2 was not significantly damaged. **c**: The numbers of follicles in each group 24 h after irradiation. **d**: The numbers of follicles in each group 72 h after irradiation. Results were presented as mean ± SEM. **p < 0.01, ***p < 0.001

As shown in Fig. 2, comparison of the ovarian tissues of rats in groups C1, R1 and S1 showed that the histological structures of the ovarian tissues in the three groups were normal with no obvious damage, and there was no significant difference in the follicle counts (*p* > 0.05). Seventy-two hours after exposure to radiation, the ovarian structure in group R2 was disordered, the morphology of the ovaries was obviously damaged by irradiation with haemorrhage in the cortex and ovarian interstitial hyperplasia, but the ovarian structure in group S2 was not extensively damaged. The follicle counts of the three groups revealed that the number of rat follicles in group R2 was significantly less than in group C2, and the number of rat follicles in group S2 was less than in group C2 but significantly more than in group R2 (*p* < 0.05).

### mRNAs profile and bioinformatics analysis

**Fig. 3:**
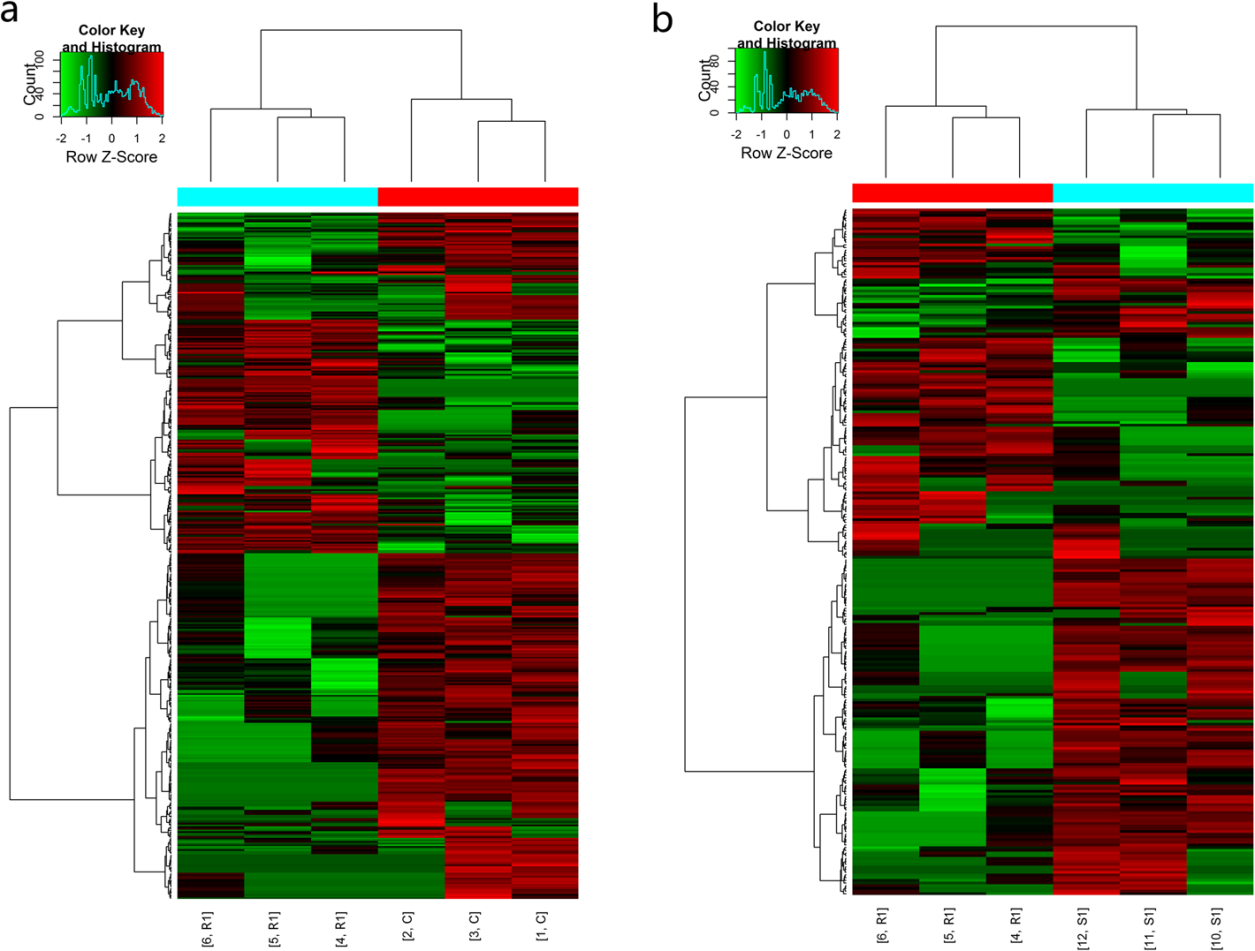
Hierarchical clustering of differentially expressed mRNAs 24 h after irradiation. **a**: radiation group compared with control group. **b**: radiation +S1P group compared with radiation group

**Fig. 4:**
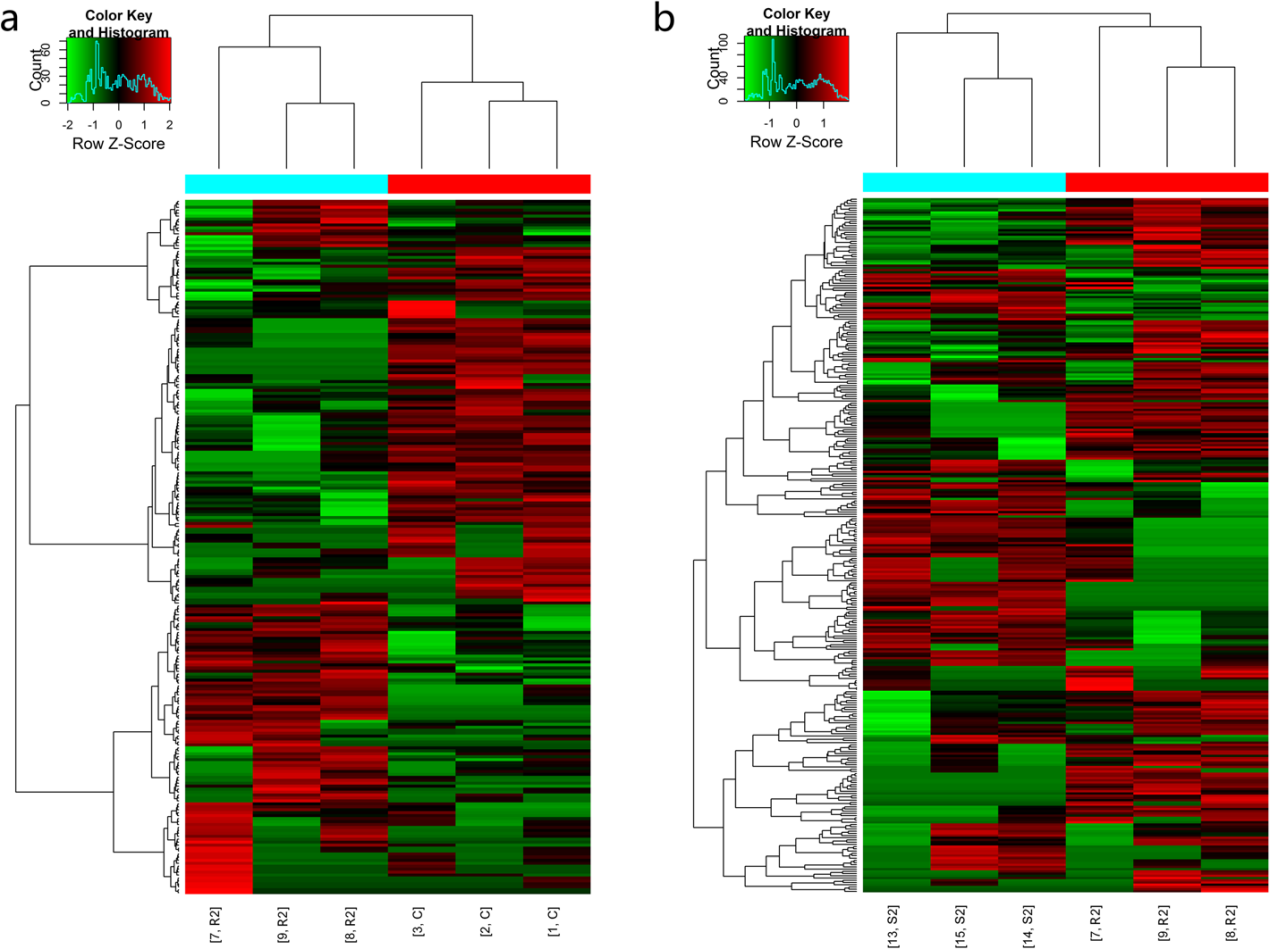
Hierarchical clustering of differentially expressed mRNAs 72 h after irradiation. **a**: radiation group compared with control group. **b**: radiation +S1P group compared with radiation group

The mRNA expression profiles of the ovarian tissues in both the radiation group and the radiation +S1P group showed significant changes 24 h and 72 h after radiation. The cluster analysis results are shown in Figs. 3 and 4. The expression of 274 mRNAs in the R1 group was downregulated and that of 143 mRNAs was upregulated 24 h after radiation when compared with the C1 group. When compared with the R1 group, the expression of 155 mRNAs was upregulated and that of 100 mRNAs was downregulated in the S1 group. Seventy-two hours after radiation, the expression of 119 mRNAs in the R2 group was downregulated and that of 116 mRNAs was upregulated when compared with the C2 group. When compared with the R2 group, the expression of 133 mRNAs was upregulated and that of 180 mRNAs was downregulated in the S2 group. The number of differentially expressed genes varied with time and S1P treatment (Table 2). A detailed list of all of these genes and their expression values is presented in Supplementary Table S1. The results of mRNA sequencing showed that among the differentially expressed genes, genes related to mitochondrial function were almost all downregulated after radiation.

**Table 2:** Number of differentially expressed genes response to radiation and S1P co-administration

24 h after radiation				72 h after radiation			
Group	Up	Down	Total	Group	Up	Down	Total
R1 vs. C1	143	274	417	R2 vs. C2	116	119	235
S1 vs. R1	155	100	255	S2 vs. R2	133	180	313

**Fig. 5:**
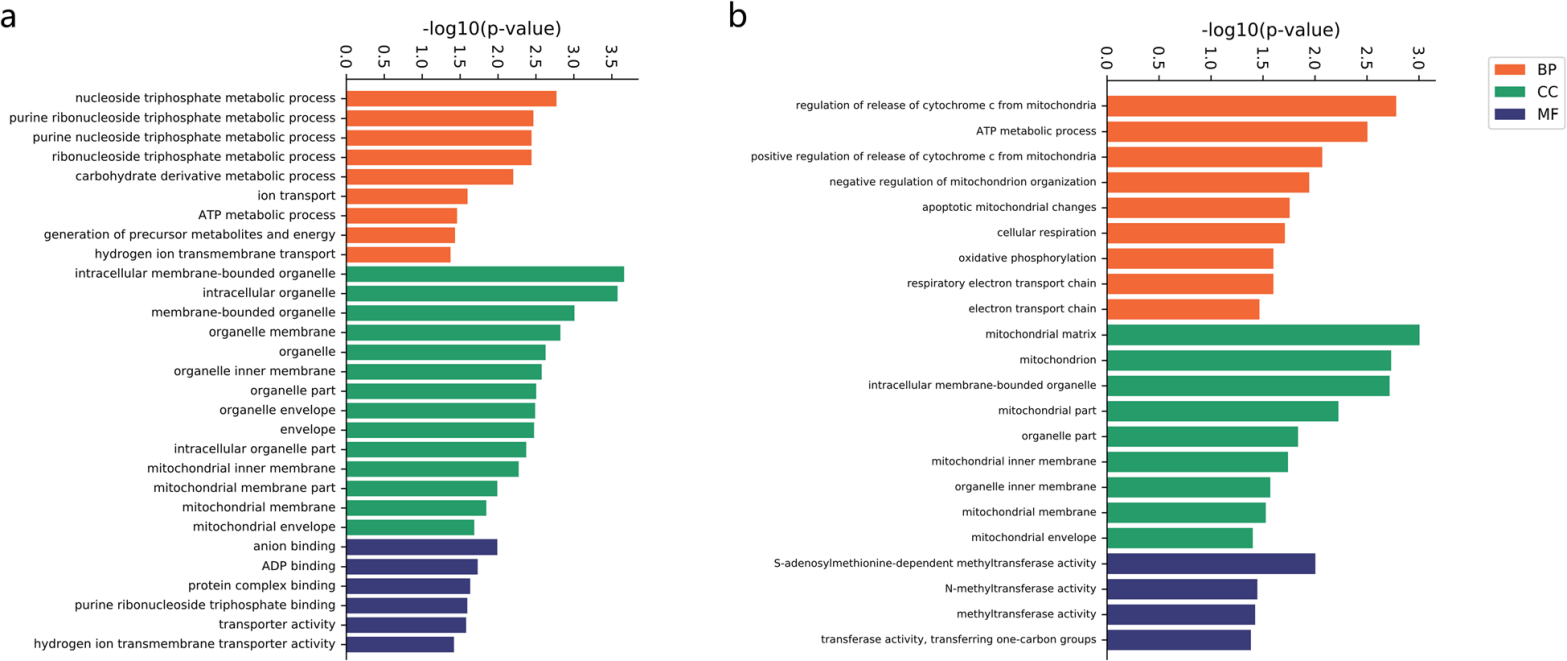
Significantly enriched GO terms related to mitochondria of differentially expressed mRNAs in all three GO categories, including Biological Process (BP), Cellular Component (CC) and Molecular Function (MF). **a**: radiation group compared with control group 24 h after irradiation. **b**: radiation group compared with control group 72 h after irradiation

**Fig. 6:**
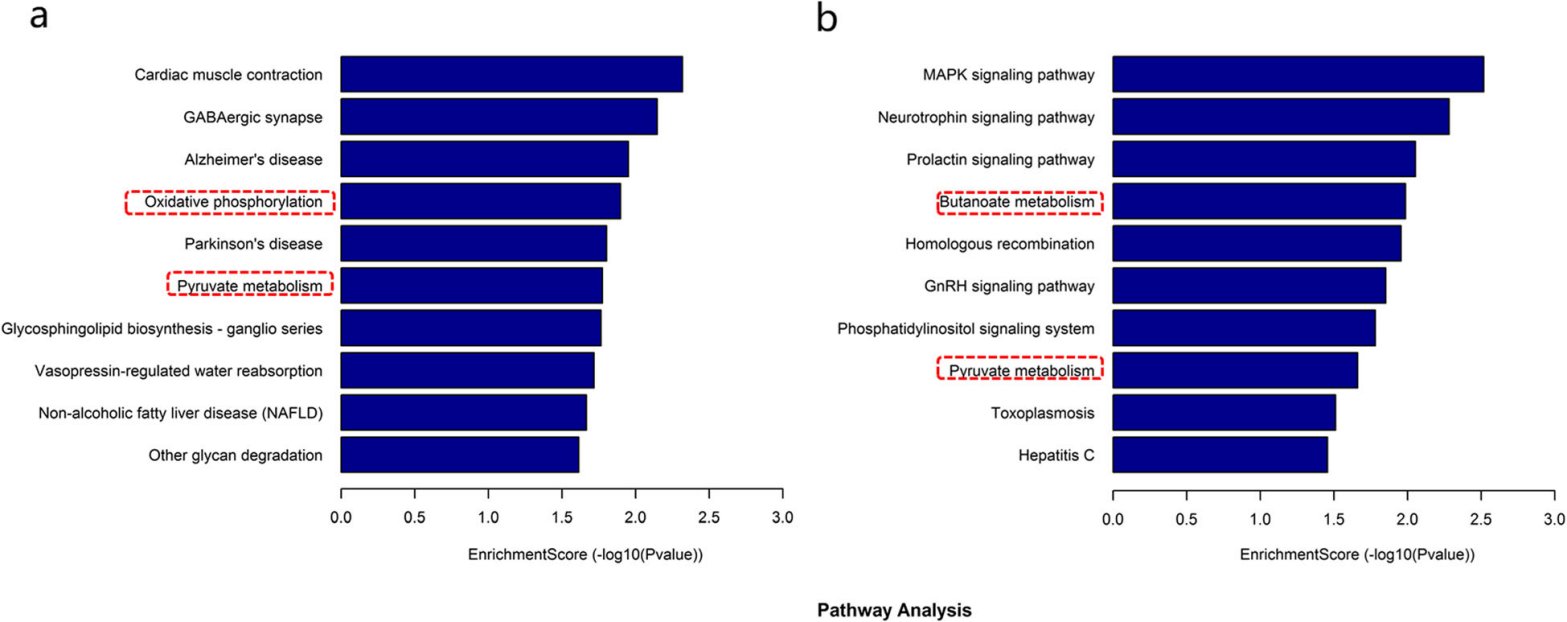
The top 10 significantly enriched pathways of decreased mRNAs expression after irradiation in KEGG pathway analysis. **a**: radiation group compared with control group 24 h after irradiation. **b**: radiation group compared with control group 72 h after irradiation. The dotted red box shows the pathways involved in mitochondrial function

We further performed gene ontology and KEGG pathway analysis of the differentially expressed mRNAs. The results of the GO enrichment revealed that biological processes related to mitochondrial function particularly were almost all downregulated after irradiation (Fig. 5). As shown in Fig. 6, KEGG pathway analysis showed that the top ten significantly enriched pathways of decreased mRNA expression in the 24- h group after radiation were cardiac muscle contraction, GABAergic synapse, Alzheimer’s disease, oxidative phosphorylation, Parkinson’s disease, pyruvate metabolism, glycosphingolipid biosynthesis-ganglio series, vasopressin-regulated water reabsorption, non-alcoholic fatty liver disease and other glycan degradation. The top ten significantly enriched pathways with downregulated mRNA expression 72 h after irradiation were MAPK signalling pathway, neurotrophin signalling pathway, prolactin signalling pathway, butanoate metabolism, homologous recombination, GnRH signalling pathway, phosphatidylinositol signalling pathway, pyruvate metabolism, toxoplasma, and hepatitis C. Of these, oxidative phosphorylation and pyruvate metabolism were also the only common pathways among the top ten pathways in the significantly enriched pathways of downregulated genes at 24 h and 72 h after irradiation, which are associated with mitochondrial function.

Considering the protective effect of S1P on ovaries, we selected mRNAs with downregulated expression in the radiation group and upregulated expression in the radiation+S1P group and screened mitochondrial-related genes through GO and KEGG analysis: UQCRH, MICU2 and GPX4 in the 24-h group and ACAT1, ALAS2, ARSB, GSR, PPIF and UQCRB in the 72-h group (Table 3).

**Table 3:** List of differentially expressed mitochondrial-related genes response to ionizing radiation

Gene	Description
24 h	
UQCRH	ubiquinol-cytochrome c reductase hinge protein
MICU2	mitochondrial Ca^2+^ uniporter protein 2
GPX4	glutathione peroxidase 4
72 h	
ACAT1	acetyl-CoA acetyltransferase 1
ALAS2	5′-aminolevulinate synthase 2
ARSB	arylsulfatase B
GSR	glutathione reductase
PPIF	peptidylprolyl isomerase F
UQCRB	ubiquinol-cytochrome c reductase binding protein

*S1P* Sphingosinol 1-phosphate

### Quantitative real-time PCR validation of mitochondrial-related genes

**Fig. 7:**
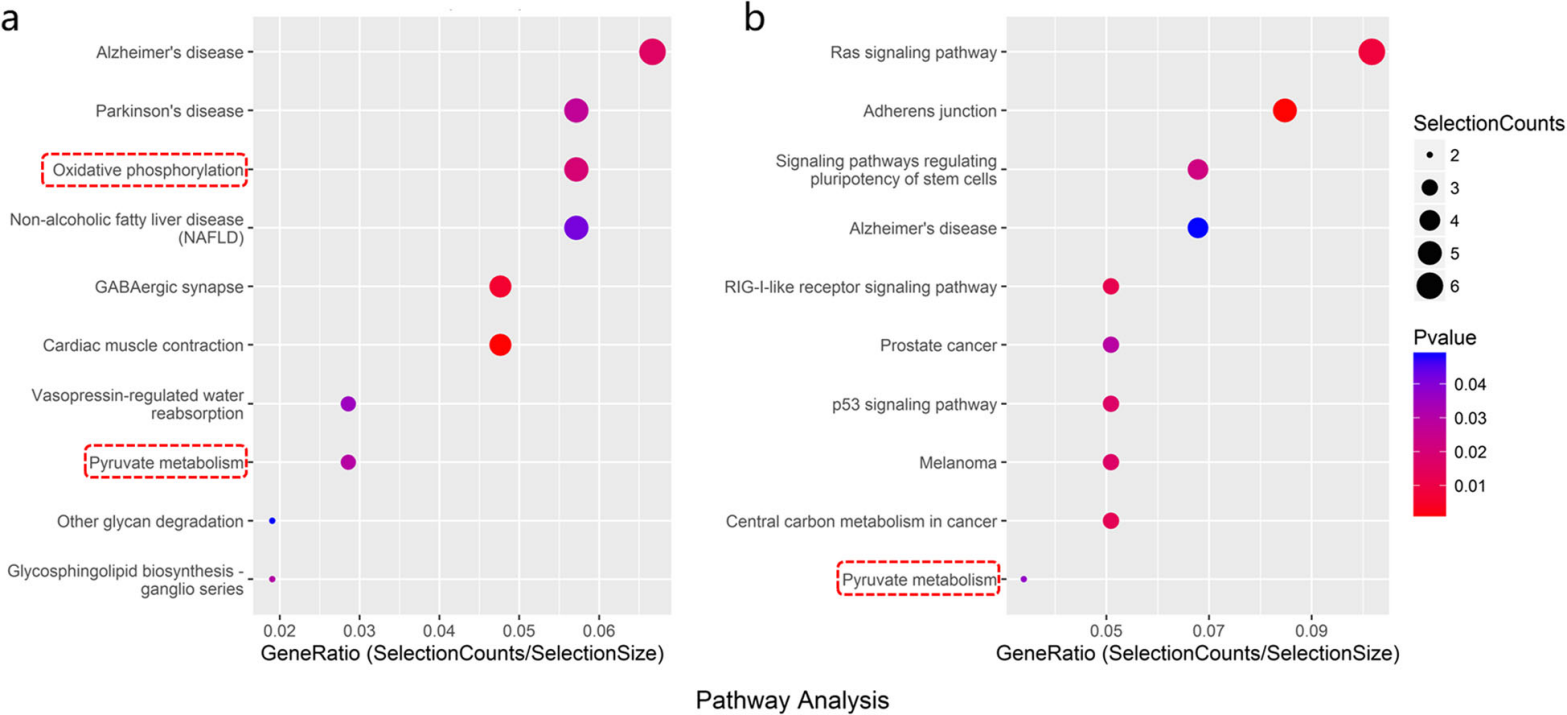
Bubble plot of the top 10 significantly enriched pathways 24 h after irradiation in KEGG pathway analysis. **a**: significantly down-regulated enriched pathways in the radiation group compared with control group **b**: significantly up-regulated enriched pathways in the radiation +S1P group compared with radiation group. The dotted red box shows the pathways involved in mitochondrial function

The mRNA sequencing results of the ovarian tissue showed that almost all of the mitochondrial-related genes were downregulated after radiation, and the administration of S1P could reverse this downward trend. As shown in Fig. 7, the KEGG pathway analysis revealed that in the radiation+S1P group, there was significant enrichment of mitochondrial function-related pathways of the upregulated genes 24 h after radiation, while there was no significantly enriched pathway of upregulated genes involved in mitochondrial function 72 h after radiation. The protective effect of S1P on mitochondria during radiation-induced ovarian injury was more obvious in the acute phase 24 h after radiation. We performed RT-PCR to validate the expression of UQCRH, MICU2 and GPX4.

**Fig. 8:**
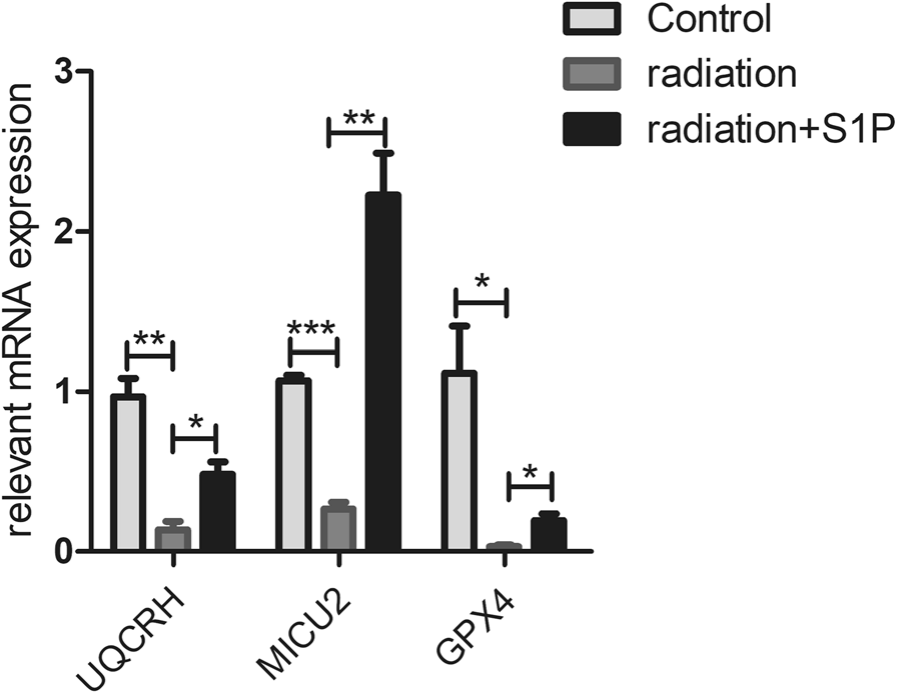
Gene expression measured by qRT-PCR. Expressions of three bi-directionally regulated genes (UQCRH, MICU2, and GPX4) screened by high-throughput sequencing were validated. ACTB was used as internal control. Results were presented as mean ± SEM. **p < 0.01, ***p < 0.001. (*n* = 3)

As shown in Fig. 8, after RT-PCR verification, the expression of UQCRH, MICU2, and GPX4 was significantly reduced 24 h after irradiation; when compared with the radiation-only group, the expression in the radiation+S1P group was significantly increased. Analysis of the gene expression of UQCRH, MICU2 and GPX4 by RT-PCR confirmed that these genes were downregulated after irradiation and reversed the direction of their changes with S1P.

## Discussion

In females, the ovaries are one of the organs that are highly sensitive to radiation. When ovaries are exposed to systemic or abdominal radiation, reproductive ageing accelerates, resulting in reduced fertility and even infertility. Damage to the ovaries from underlying radiation includes oocyte death, granulosa cell damage, and stromal damage [14]. Oocytes are extremely radiosensitive, and follicular reserve depletion due to exposure to radiation may lead to premature ovarian failure, early menopause, and infertility [3]. Granulosa cells with mitotically activity will die within only a few hours of exposure to radiation, while nearly half of immature oocytes will undergo structural damage when exposed to 2Gy of irradiation [4]. Therefore, how to ameliorate the damage to female reproductive system function following ionizing radiation has become a research hotspot.

The overall aim of this study was to further verify the damaging effects of radiation on ovarian tissue and the protective effect of S1P against it and to investigate whether mitochondrial-related genes are involved in these processes. In this study, SD female rats were treated with exposure to X-ray radiation and S1P co-administration starting 72 h before radiation until they were sacrificed. The serum AMH levels, ovarian histological changes and follicular counts were detected 24 h and 72 h after radiation, and mRNA expression changes in ovarian tissues were detected by high-throughput sequencing technology. The results showed that the number of follicles was significantly decreased and the serum AMH level was reduced after the ovary was exposed to 2Gy X-ray radiation, which indicates that the ovarian function and fertility of female SD rats were significantly impaired. Serum AMH levels are directly associated with a decrease in ovarian follicle numbers and have been described as one of the most reliable and accurate markers of ovarian reserve function [15]. However, administration of S1P prevented the decrease in AMH levels when compared to the radiation groups and alleviated the ovarian histomorphological disorder caused by radiation, which showed less haemorrhage in the cortex, less interstitial fibrosis and more follicles with intact oocytes, indicative of the radioprotective effect of S1P on the ovaries. Through sequencing analysis, we found that there were significant differences in the mRNA expression profile between the radiation group and the S1P pretreated group, and the differentially expressed mRNAs were significantly diverse 24 and 72 h after irradiation. GO and KEGG pathway analysis revealed that mitochondria played a role in both ovarian radiation injury and the protective effect of S1P on radiation-induced injury, but with the passage of time after radiation, the genes involved were different. Moreover, the protective effect of S1P on mitochondria in radiation-induced ovarian injury was more obvious in the acute phase 24 h after radiation.

S1P is a kind of biologically active polar lipid. Several studies have demonstrated that S1P can regulate cell proliferation, growth, and survival [6]. Previous studies have also revealed that S1P could protect granulosa cells and oocytes from radiation damage and attenuate the apoptosis of granulosa cells after hydrogen peroxide treatment [16]. A study by Zelinski Mary B et al. showed that female macaques’ ovaries rapidly failed after 15 Gy radiation, while with S1P or its analogue, fingolimod (FTY720) pretreatment, macaques would quickly resume menstruation and fertility was restored after radiation, and their offspring developed normally without genomic instability [17]. Currently, the active metabolite fingolimod-phosphate (FP), one of the oral drugs for the treatment of multiple sclerosis (MS), seems to increase the stability of mitochondria in neurons, regulate mitochondrial oxidative stress, and restore mitochondrial kinetics under oxidative stress [10]. These characteristics of S1P and its analogues make it one of the most promising protective agents for radiation-induced ovary damage in the future. Therefore, understanding the precise protective mechanism of S1P is becoming intensively important to provide a theoretical basis for the future application of S1P and its analogues in radiation protection, as well as providing a foundation for further research on radiation protection.

Mitochondria, known as the “power factory” of cells that provide more than 95% of the energy for the body, are the main sites of oxidative phosphorylation and ATP formation in cells. Mitochondria are also the main source of endogenous oxygen free radicals, participating in diverse cellular functions including cell apoptosis, signal transduction and regulation, and are associated with a variety of diseases [18]. Ionizing radiation can cause alterations in mitochondrial dynamics and increase the level of intracellular reactive oxygen species (ROS) through mitochondrial dysfunction, but the mechanism involved in mitochondrial dysfunction is still unclear [19].

As mentioned above, the expression of mitochondrial-related genes UQCRH, MICU2 and GPX4 in ovarian tissues was downregulated 24 h after irradiation, while their expression was upregulated in the S1P group when compared with the radiation-only group. Ubiquinol cytochrome c reductase hinge (UQCRH), located in the mitochondrial inner membrane, is the main subunit of mitochondrial respiratory chain complex III. In the process of oxidative phosphorylation, UQCRH is responsible for the electron transfer between cytochrome C and cytochrome C1 and is part of the most critical step in the electron transport cascade, which is involved in the oxidative phosphorylation of mitochondria. UQCRH is highly conserved from yeast to humans and plays a critical role in cell ageing [20]. The mitochondrial Ca2+ uptake 2 (MICU2), located in the mitochondria, is a major component of the mitochondrial calcium uniporter complex. As the Ca^2+^ sensing protein in the mitochondrial unidirectional transporter, it is a highly selective Ca^2+^ channel that mediates mitochondrial Ca^2+^ uptake to regulate cell death, metabolism, and cytoplasmic Ca^2+^ signalling. Mitochondria are one of the most important intracellular organelles for the regulation of Ca^2+^ homeostasis due to their strong Ca^2+^ buffering capacity. The regulation of mitochondrial Ca^2+^ is crucial for biological functions and cell signal transduction [21]. The mitochondrial calcium unidirectional transport complex plays an essential role in mitochondrial calcium homeostasis. Existing studies have suggested that MICU2 deficiency damages mitochondrial Ca^2+^ homeostasis. In the nervous system, invalid mutations in the MICU2 gene lead to abnormal mitochondrial calcium homeostasis and severe neurodevelopmental disorders [22]. Therefore, our study supports the idea that mitochondrial dysfunction and calcium homeostasis may be critical in the process of radiation-induced ovarian injury and may also be involved in the radioprotective effect of S1P. Glutathione peroxidase 4 (GPX4) is the only known major antioxidant enzyme directly reducing phospholipid hydroperoxide in membranes and lipoproteins, which could inhibit lipid peroxidation and has a variety of biological functions, including sperm maturation, apoptosis regulation and brain embryogenesis [23]. After cell or body exposure to radiation, mitochondrial function is impaired, ROS levels increase, and lipid peroxidation occurs. Overexpression of GPX4 has been shown to reduce ischaemia/reperfusion-related cardiac dysfunction [24]. In the male reproductive system, GPX4 levels are correlated with fertility-related parameters. Previous studies have found that the decline in GPX4 is related to radiation-induced ferroptosis, which may play an important role in acute radiation-induced lung injury (RILI) [25]. Hence, it cannot be ruled out that ferroptosis may participate in radiation-induced ovarian injury.

The expression of the UQCRH, MICU2 and GPX4 genes was significantly downregulated after radiation, indicating that the mechanisms of radiation damage to ovarian function include inhibition of the mitochondrial oxidative respiratory chain, abnormal calcium homeostasis and augmented oxidative stress. The proton gradient across the mitochondrial inner membrane produced by the mitochondrial oxidative respiratory chain is necessary to maintain normal mitochondrial calcium flow. Abnormal mitochondrial calcium homeostasis can accelerate the production of reactive oxygen species (ROS). Therefore, a positive feedback regulatory loop is formed among the mitochondrial calcium influx-respiratory chain-proton gradient across the mitochondrial inner membrane, and the three are closely related, thus exacerbating the damage effects [21]. Therefore, the downregulation of UQCRH, MICU2 and GPX4 genes after radiation may have a deeper connection and synergistic effect on the damage to mitochondrial function. This study showed that the protective effect of S1P on mitochondria was more significant 24 h after radiation, which we speculated might be related to the generation of mitochondrial dysfunction and the time of ROS production after radiation. The study by Takakoyoshida et al. showed that mitochondrial dysfunction occurred within 12 h after radiation and lasted up to 72 h, and ROS production and mitochondrial DNA oxidation occurred within 24 h after radiation [26]. The significant increase in ROS levels in mitochondria may be caused by the electron leakage of the mitochondrial complex. In this study, UQCRH, GPX4, and MICU2, which are involved in the composition of respiratory chain complexes, inhibition of oxidative stress and regulation of Ca2+ homeostasis, significantly decreased 24 h after irradiation, while S1P significantly reduced this trend. S1P can protect ovarian function by alleviating the early generation of oxidative stress after radiation. In this study, we found significant changes in mitochondrial-related genes 24 and 72 h after radiation and verified the protective effect of S1P on radiation-induced ovarian damage. Therefore, we did not conduct a longer duration experiment to investigate the variation tendency of mitochondrial-related genes after 72 h of radiation exposure, which is also a limitation of this study.

To the best of our knowledge, this is the first study focusing on the role of mitochondrial-related genes in radiation-induced ovarian injury and the potential radioprotective effect of S1P. Our study found that mitochondrial-related genes were differentially expressed 24 h and 72 h after radiation, but the differentially expressed genes were remarkably distinct at different time points. However, the protective effect of S1P on mitochondria in the radiation-induced ovarian injury was more pronounced 24 h after radiation. Certainly, additional studies are needed to further examine the role of these differentially expressed genes related to mitochondria in the protection of S1P against gonadotoxic actions caused by irradiation.

## Conclusion

The ovarian function of SD female rats is significantly decreased after being exposed to 6-MV X-ray with a total dose of 2Gy. S1P has a protective effect against radiation-induced ovarian injury, and mitochondria may play a critical role in this. The specific mechanisms of the mitochondria that participated in radiation-induced ovarian injury and the potential radioprotective effect of S1P still need to be further explored in future.

## Supplementary information

**Additional file 1 Table S1**. Detailed list of all these differentially expressed genes and their expression values.

## Data Availability

Data are available upon request from the corresponding author.

## Abbreviations

S1P: Sphingosine 1-phosphate
AMH: Anti-Müllerian hormone
KEGG: Kyoto Encyclopedia of Genes and Genomes
GO: Gene ontology
RT-PCR: Real-time quantitative polymerase chain reaction
UQCRH: Ubiquinol cytochrome c reductase hinge
MICU2: Mitochondrial Ca2+ uptake 2
GPX4: Glutathione Peroxidase 4
ROS: Reactive oxygen species
