# Supplementary material for: Sphingosine 1-phosphate protects against radiation-induced ovarian injury in female rats—impact on mitochondrial-related genes
Source: Reprod Biol Endocrinol. 2020 Oct 12;18:99. doi: 10.1186/s12958-020-00659-z (PMC7549217; doi:10.1186/s12958-020-00659-z)
Supplement: Supplementary file 1 — Additional file 1 Table S1. Detailed list of all these differentially expressed genes and their expression values. [file 12958_2020_659_MOESM1_ESM.docx]

Table S1：Detailed list of all these differentially expressed genes and their expression values

| **24h after radiation** | | | | | | **72h after radiation** | | | | | |
| --- | --- | --- | --- | --- | --- | --- | --- | --- | --- | --- | --- |
| **R1 vs.C1** | | | **S1 vs.R1** | | | **R2 vs.C2** | | | **S2 vs.R2** | | |
| **Up-regulated genes** | **Log FC** | ***P*** | **Up-regulated genes** | **Log FC** | ***P*** | **Up-regulated genes** | **Log FC** | ***P*** | **Up-regulated genes** | **Log FC** | ***P*** |
| Akr1e2 | 3.032148 | 0.016982 | Psme1-ps1 | 2.370353 | 0.012316 | Eci2 | 3.270717 | 1.32E-05 | Hyal1 | 3.144454 | 0.017255 |
| Slfn2 | 2.463149 | 0.035154 | Hyal1 | 3.124596 | 0.034394 | Maoa | 2.355858 | 0.044659 | Emcn | 2.567529 | 0.019136 |
| Mab21l2 | 3.667548 | 0.019771 | Dlgap1 | 2.070841 | 0.038255 | Ahcyl2 | 2.772377 | 0.034003 | Blmh | 5.316893 | 0.031045 |
| Lsp1 | 2.834737 | 0.049464 | Mef2b | 2.762326 | 0.021562 | Cfap65 | 2.483923 | 0.027954 | Cpt1a | 2.000519 | 0.046865 |
| Tfcp2 | 2.577867 | 0.044112 | Ech1 | 2.607072 | 0.014623 | Bcl2a1 | 2.849936 | 0.044632 | Parp2 | 2.821076 | 0.001338 |
| Cryz | 5.170191 | 0.026306 | Ctage5 | 2.094909 | 0.04412 | Zbtb47 | 3.828323 | 0.007531 | AABR07000159.2 | 6.213321 | 0.002989 |
| Trmt44 | 2.317548 | 0.047748 | AABR07000159.2 | 6.01141 | 0.006623 | Ackr4 | 2.125527 | 0.029106 | Pla2g2a | 6.020996 | 0.005317 |
| Eci2 | 2.492518 | 0.000553 | Rragc | 3.806516 | 0.0101 | Nrcam | 3.57021 | 8.53E-06 | Uqcrb | 2.54207 | 0.049478 |
| Chrna2 | 1.763035 | 0.016022 | Ankrd10 | 5.330309 | 0.038241 | Pdzd4 | 2.288055 | 0.04174 | Mettl9 | 2.863037 | 0.049107 |
| Maoa | 2.579789 | 0.026647 | ST7 | 1.783697 | 0.034831 | Hint3 | 5.589836 | 0.031083 | Rusc2 | 3.174706 | 0.049048 |
| Vsir | 3.12174 | 0.014883 | Nsrp1 | 1.675353 | 0.044579 | Polr2l | 4.108789 | 0.003019 | Casp6 | 2.661069 | 0.032993 |
| Wdr4 | 1.882283 | 0.022057 | Ptprm | 3.12231 | 0.038001 | Tlr3 | 2.720917 | 0.00184 | Rnf34 | 2.193861 | 0.041463 |
| Ahcyl2 | 2.776519 | 0.033808 | Wbp2 | 2.909039 | 0.009163 | Nell1 | 2.910597 | 0.000112 | Asph | 1.617321 | 0.024149 |
| Gls | 1.936263 | 0.007722 | Id4 | 5.797627 | 0.00764 | Cpxm1 | 2.001256 | 0.048082 | Lage3 | 5.929149 | 0.004262 |
| Slc5a6 | 3.352196 | 0.04087 | Itm2c | 1.73872 | 0.03245 | Nbl1 | 2.804944 | 0.035199 | AABR07051548.1 | 3.421234 | 0.000738 |
| Wbp2nl | 2.135441 | 0.039366 | Csrp2 | 2.380885 | 0.039212 | Lgals3bp | 2.04237 | 0.028221 | Rpl21 | 2.58698 | 0.022887 |
| Map7 | 1.70926 | 0.022531 | Midn | 2.279325 | 0.036217 | Prkag2 | 3.483773 | 1.58E-05 | Dgkz | 2.867635 | 0.041317 |
| LOC103691261 | 2.847366 | 0.032679 | RGD1311345 | 2.828209 | 0.036033 | RT1-CE10 | 1.867587 | 0.046639 | Chchd6 | 3.630276 | 0.030702 |
| Ackr4 | 2.004828 | 0.039094 | AABR07024439.1 | 3.744543 | 0.00276 | Wdfy3 | 2.363785 | 0.03228 | LOC257642 | 1.549236 | 0.043304 |
| Fam71f2 | 6.457337 | 0.003313 | Sugt1 | 1.971925 | 0.036208 | Rsph1 | 2.342874 | 0.03289 | Nabp2 | 2.214227 | 0.029476 |
| Exph5 | 1.776377 | 0.025554 | Cfap44 | 2.064271 | 0.045599 | Akr1c1 | 2.878623 | 0.040695 | Foxo3 | 3.072446 | 0.031702 |
| Nrcam | 2.153665 | 0.004582 | Polr2l | 3.338045 | 0.006483 | RGD1309594 | 2.548045 | 0.048429 | Parn | 3.25159 | 0.022516 |
| Zfp703 | 2.665077 | 0.040077 | Apmap | 2.689767 | 0.036665 | Cnn1 | 3.118094 | 0.001041 | Atad3a | 2.633171 | 0.033073 |
| Abcc9 | 2.290989 | 0.02583 | Ptprcap | 3.168349 | 0.004152 | Msmb | 2.686717 | 0.037177 | Bfar | 3.699024 | 0.003115 |
| Nell1 | 1.763718 | 0.014222 | Arpin | 2.262642 | 0.027627 | Cpne2 | 2.281833 | 0.036945 | Acat1 | 2.557023 | 0.00385 |
| Tmem108 | 2.836864 | 0.0023 | Rap1gds1 | 2.26997 | 0.042063 | Sun1 | 2.748371 | 0.049783 | Pla2g1b | 2.117678 | 0.043265 |
| Tex12 | 1.767763 | 0.019609 | Mas1 | 1.664619 | 0.042539 | Stap1 | 2.810996 | 0.049574 | Fcgr1a | 3.730249 | 0.01965 |
| Slfn13 | 1.865173 | 0.046946 | LOC103689927 | 3.910852 | 0.01059 | Cfap70 | 2.710403 | 0.043337 | Tmem97 | 5.04298 | 0.041479 |
| LOC497938 | 5.355141 | 0.036685 | Usp5 | 3.53769 | 0.018363 | Notch1 | 3.261613 | 0.034387 | Colq | 3.440454 | 0.041738 |
| AABR07068650.1 | 2.135652 | 0.047568 | Chn1 | 5.795021 | 0.005577 | Lrrc51 | 2.958564 | 0.002288 | Olr1433 | 4.850304 | 0.019402 |
| RT1-CE10 | 2.304714 | 0.013636 | Acat1 | 1.986873 | 0.025011 | Ehd3 | 3.314592 | 0.037793 | Tex15 | 3.768671 | 0.008759 |
| Rab25 | 3.341251 | 0.03995 | Myo1b | 3.442735 | 0.022011 | Cebpd | 3.726973 | 0.014195 | Grin1 | 2.226558 | 0.002501 |
| Gjb6 | 2.886152 | 0.031205 | Fam26d | 2.681251 | 0.008565 | Srgap2 | 2.657417 | 0.035922 | St5 | 3.520002 | 0.005365 |
| Mybpc1 | 2.444997 | 0.030507 | RGD1359290 | 3.768233 | 0.001446 | Calr3 | 2.269603 | 0.027349 | Rab36 | 2.312275 | 0.045715 |
| Rps6kb2 | 5.66388 | 0.018124 | Bptf | 3.383893 | 0.001936 | Mef2d | 5.063711 | 0.035216 | Pole4 | 3.004652 | 0.042252 |
| Wdfy3 | 2.547291 | 0.020128 | Krt19 | 2.407026 | 0.026573 | Ptgds | 2.417604 | 0.032088 | Recql5 | 2.018934 | 0.014615 |
| Cc2d1a | 2.539114 | 0.001311 | Derl1 | 2.472479 | 0.049145 | Ppil4 | 2.741548 | 0.047368 | Fbxo2 | 3.574834 | 0.006988 |
| Msmb | 3.518226 | 0.005875 | Padi2 | 5.020705 | 0.027687 | Etnk1 | 2.986417 | 0.046342 | Lmo2 | 3.479712 | 0.012979 |
| RGD1565693 | 1.948034 | 0.036756 | Nrip1 | 3.002117 | 0.042623 | Fastkd1 | 2.486259 | 0.018446 | AC121204.1 | 2.68666 | 0.047439 |
| Fpr1 | 3.639737 | 0.039486 | LOC103689986 | 1.718026 | 0.022615 | Senp17 | 2.712263 | 0.009429 | Nptx2 | 2.829892 | 0.035745 |
| Tbata | 2.346349 | 0.042254 | Uba6 | 3.928098 | 0.006023 | Golga7 | 4.68991 | 3.94E-05 | Ndufa11 | 2.786084 | 0.002124 |
| Slc17a2 | 3.968137 | 0.009805 | Pkm | 5.483696 | 0.018991 | Myo5b | 3.443937 | 0.009251 | Etfb | 2.171074 | 0.027989 |
| Lrrc51 | 2.240827 | 0.020362 | Rap2a | 5.06483 | 0.041627 | Ttc22 | 2.291184 | 0.003687 | Dpy30 | 2.743155 | 0.044621 |
| Apobr | 1.535997 | 0.032408 | Snapc2 | 2.766198 | 0.037833 | Fam124a | 5.003474 | 0.034325 | Igsf11 | 2.237507 | 0.005274 |
| Lsm1 | 2.025613 | 0.026207 | Zbtb8a | 3.786193 | 0.009857 | Rnd3 | 2.760205 | 0.046981 | Tmprss11a | 2.770933 | 0.042251 |
| AABR07020999.1 | 2.356882 | 0.004633 | Uqcrq | 3.399209 | 0.007155 | Llgl1 | 1.641225 | 0.036949 | Rpl39l | 2.838166 | 0.023641 |
| LOC102555635 | 3.05818 | 0.04671 | Scyl2 | 2.390722 | 0.045894 | Sept12 | 1.84898 | 0.029928 | Fam3c | 2.219648 | 0.017347 |
| Akr1c12l1 | 2.516396 | 0.004687 | Hddc2 | 5.382152 | 0.037919 | Tns1 | 2.226325 | 0.018612 | Mysm1 | 2.919255 | 0.045627 |
| Limd2 | 2.84472 | 0.04613 | Smim15 | 2.171627 | 0.043946 | Akr1c19 | 2.062539 | 0.005012 | Atp6v1g2 | 5.479384 | 0.019744 |
| Srgap2 | 3.18131 | 0.011586 | Abca1 | 2.305544 | 0.010106 | Maf | 5.950568 | 0.002537 | LOC103690170 | 2.38265 | 0.026438 |
| Batf3 | 2.239255 | 0.006732 | Jdp2 | 5.568978 | 0.022673 | Rgs9 | 3.477843 | 0.019666 | LOC257642 | 2.687764 | 0.00111 |
| Grasp | 3.473882 | 0.042796 | Ptprf | 2.124309 | 0.026294 | Plekhm2 | 3.130312 | 0.038704 | LOC500300 | 3.131218 | 0.03981 |
| Gcnt2 | 3.274595 | 0.002958 | C1qtnf3 | 3.055263 | 0.049949 | Krt15 | 3.520729 | 0.004267 | Maats1 | 2.757026 | 0.001834 |
| Galnt11 | 2.798292 | 0.0378 | Rbm7 | 5.095479 | 0.026784 | Timp3 | 3.481822 | 0.001481 | Iah1 | 3.193612 | 0.041396 |
| Tas1r3 | 2.381336 | 0.010407 | Cxcl14 | 3.509951 | 0.006342 | Abl2 | 2.455521 | 0.015359 | G3bp1 | 2.058851 | 0.035321 |
| Ncoa6 | 3.374266 | 0.008392 | Acot6 | 3.383303 | 0.033765 | Tmem39a | 5.149077 | 0.035517 | Rpl27 | 2.449207 | 0.040441 |
| P2ry14 | 2.986505 | 0.034844 | Arf1 | 2.42305 | 0.012891 | Fgf10 | 3.156493 | 0.038562 | Adipoq | 2.845941 | 0.015059 |
| Etnk1 | 3.530847 | 0.018097 | Sec31a | 3.258285 | 0.046715 | Slc22a12 | 3.620572 | 0.021999 | Ptprc | 3.238703 | 0.04159 |
| Prkag3 | 3.006944 | 0.014402 | Ptgds | 3.033742 | 0.010089 | Tsx | 3.603502 | 0.031415 | Mcrs1 | 3.600133 | 0.015187 |
| Etnk2 | 2.877632 | 0.010026 | Ifih1 | 2.530706 | 0.021776 | Elf3 | 1.846138 | 0.044306 | Msl3 | 3.529278 | 0.035657 |
| Fastkd1 | 2.491489 | 0.017696 | Lmo3 | 2.559175 | 0.049956 | Cygb | 5.303361 | 0.021638 | Snrpd1 | 5.689914 | 0.023132 |
| B3gnt9 | 3.621488 | 0.025332 | Unc80 | 5.262219 | 0.04993 | Eps15l1 | 2.141207 | 0.043484 | Clec10a | 3.608562 | 0.0299 |
| Ccdc122 | 2.035625 | 0.032029 | Lmnb2 | 3.278686 | 0.036828 | Pes1 | 2.102 | 0.041834 | S100g | 3.926788 | 0.00125 |
| Ccnc | 5.549617 | 0.01376 | Ptbp1 | 6.238583 | 0.000704 | B3gnt7 | 4.704236 | 0.045952 | Zfp706 | 2.131299 | 0.010321 |
| C1rl | 1.667708 | 0.019391 | Slitrk6 | 5.544448 | 0.026422 | Acan | 1.620755 | 0.026938 | Arsb | 3.436914 | 0.006669 |
| Serp2 | 2.172755 | 0.038436 | Wasf2 | 4.030774 | 0.002761 | Clec16a | 4.255934 | 0.000154 | Zfp57 | 1.975115 | 0.015895 |
| Senp17 | 3.273128 | 0.001603 | Znrf2 | 2.691543 | 0.01884 | Adam7 | 2.518786 | 0.009256 | Rasal1 | 3.516855 | 0.019471 |
| Golga7 | 3.323185 | 0.003681 | Il6st | 5.580734 | 0.01045 | AABR07071287.1 | 2.336338 | 0.041367 | LOC100911794 | 1.783116 | 0.027814 |
| Ttc22 | 2.538261 | 0.001273 | Lin37 | 5.261751 | 0.049288 | Irf6 | 2.620282 | 0.049835 | Ninj2 | 3.144971 | 3.64E-05 |
| Fam124a | 5.605581 | 0.011579 | Atxn10 | 5.597065 | 0.010326 | RGD1565536 | 3.667241 | 0.025045 | Swi5 | 3.101591 | 0.022725 |
| Kctd17 | 2.838058 | 0.037956 | Aph1a | 2.340577 | 0.031838 | RGD1562618 | 3.305811 | 0.021216 | Bysl | 5.50463 | 0.016081 |
| Xrra1 | 2.825955 | 0.029866 | Cdk16 | 3.706548 | 0.00915 | Lrrc14 | 1.821538 | 0.049472 | Ifrd2 | 3.210945 | 4.88E-05 |
| Sept12 | 2.31025 | 0.006537 | Hbb | 3.574197 | 0.014475 | Ssx1 | 1.888654 | 0.045267 | Wwox | 2.546215 | 0.031386 |
| Ercc3 | 5.258702 | 0.024212 | Zrsr2 | 2.971291 | 0.013558 | Klhl35 | 3.34471 | 0.039089 | Fyco1 | 2.388019 | 0.030462 |
| Zcchc2 | 2.273312 | 0.048371 | Psmc4 | 3.725737 | 0.005817 | Ube2n | 4.773678 | 0.047993 | Tmem30c | 3.466135 | 0.033261 |
| Slc35e1 | 2.41199 | 0.038034 | Tnks1bp1 | 2.349729 | 0.049082 | Fam13a | 2.155411 | 0.043738 | Ctif | 2.042101 | 0.044559 |
| Akr1c19 | 3.27509 | 1.69E-05 | Slc6a6 | 2.849067 | 0.013095 | Acsl3 | 2.221214 | 0.042094 | Cep85l | 1.746908 | 0.049141 |
| Maf | 5.896653 | 0.002772 | S100g | 3.025667 | 0.029898 | Slco2b1 | 3.049504 | 0.016725 | Tmprss2 | 2.035007 | 0.005492 |
| Dr1 | 6.248149 | 0.005476 | Casp8 | 2.482404 | 0.013794 | Htra4 | 1.647065 | 0.049982 | Adcy10 | 2.802788 | 0.034 |
| Dmkn | 1.636895 | 0.030906 | Ap3s1 | 2.987478 | 0.02914 | Mogat1 | 2.221972 | 0.001782 | Polr2f | 4.069964 | 0.009166 |
| Adgrf5 | 3.01747 | 0.043776 | Shoc2 | 3.241084 | 0.045649 | Ifit3 | 3.136175 | 0.043674 | Trim7 | 2.428092 | 0.025694 |
| Ebf3 | 5.264297 | 0.03322 | Dad1 | 3.37001 | 0.015624 | Ccl12 | 2.991892 | 0.043675 | Pip5k1a | 3.268893 | 0.004573 |
| Mmp20 | 2.316553 | 0.014047 | Kcnj8 | 3.225597 | 0.040901 | Skap2 | 3.350792 | 0.027409 | Selp | 2.977155 | 0.038214 |
| Atp6v1f | 2.812154 | 0.039743 | Uqcrh | 2.007296 | 0.026957 | Mical2 | 2.759148 | 0.02794 | Rsph3 | 3.589967 | 0.008745 |
| Abl2 | 2.275421 | 0.023909 | Bysl | 3.416148 | 0.024327 | Rnf166 | 2.018684 | 0.022995 | Rwdd2a | 2.053554 | 0.018266 |
| Cd59 | 2.225907 | 0.013315 | Ifrd2 | 3.711878 | 3.88E-05 | Prob1 | 3.562117 | 0.021489 | Alas2 | 4.047629 | 0.006437 |
| Col9a2 | 1.560026 | 0.041479 | Ankfy1 | 2.658515 | 0.038309 | Gpr108 | 5.361843 | 0.033111 | Ctr9 | 1.892305 | 0.020723 |
| Syce1l | 3.431917 | 4.65E-05 | Gramd1a | 2.200873 | 0.027354 | Adam30 | 3.043615 | 0.01034 | Tep1 | 2.199687 | 0.03375 |
| Me3 | 2.089863 | 0.003414 | Izumo1 | 2.67762 | 0.008845 | Dhx38 | 2.706258 | 0.014026 | RGD1561231 | 1.503188 | 0.038939 |
| Ptpn1 | 5.28868 | 0.027618 | RGD1559904 | 4.900211 | 0.033152 | LOC498933 | 2.264968 | 0.025699 | RGD1560860 | 3.299629 | 0.04067 |
| RT1-CE16 | 2.239162 | 0.007402 | Tmem151b | 2.142053 | 0.017897 | Magt1 | 2.880575 | 0.03261 | Wdr74 | 5.356906 | 0.019385 |
| Cygb | 4.917293 | 0.038832 | Selt | 5.033214 | 0.042393 | Shc4 | 1.451345 | 0.048873 | Pld3 | 3.800932 | 0.00799 |
| Smagp | 2.00034 | 0.039214 | Fgfr1 | 2.736346 | 0.03256 | Nfkb1 | 2.803396 | 0.015045 | Sgpp1 | 3.18955 | 0.040445 |
| Tmprss2 | 3.931158 | 6.89E-07 | Lamtor5 | 3.136646 | 0.020795 | Zhx2 | 1.564774 | 0.04622 | Pi4k2b | 3.177698 | 0.044821 |
| Ccnf | 3.551055 | 0.03341 | Slc4a7 | 3.232332 | 0.047196 | Eif2b4 | 2.86345 | 0.026429 | Wnt5b | 3.061712 | 0.045626 |
| Eps15l1 | 2.21006 | 0.03622 | Igf1 | 2.52588 | 0.030787 | Fahd2a | 3.941117 | 0.013302 | Svip | 3.361847 | 0.004308 |
| LOC100912373 | 3.587242 | 0.002426 | Ogt | 2.405375 | 0.029069 | Akr1c13 | 1.462349 | 0.034233 | Lhcgr | 2.870466 | 0.014069 |
| Sirt7 | 2.863814 | 0.042372 | Adam7 | 2.040571 | 0.014884 | Styxl1 | 3.376631 | 0.014235 | Phf21a | 2.914054 | 0.042265 |
| Lhpp | 2.469457 | 0.017977 | Tomm22 | 2.531353 | 0.021749 | LOC100912849 | 5.598461 | 0.012666 | Mknk2 | 5.289303 | 0.021353 |
| Pipox | 3.333927 | 0.031645 | Jkamp | 3.15541 | 0.038297 | Ccdc96 | 2.073754 | 0.038622 | Mtmr2 | 2.707963 | 0.045633 |
| Synj2 | 3.184759 | 0.043591 | AABR07071287.1 | 3.31003 | 0.016371 | LOC100302465 | 3.051269 | 7.68E-05 | Scnn1a | 3.293087 | 0.049393 |
| Acan | 1.883583 | 0.010043 | Rsph3 | 3.703913 | 0.011383 | Lypla2 | 2.61971 | 0.025356 | Ndufs8 | 3.065482 | 0.032761 |
| Adam7 | 2.051561 | 0.034096 | Hps1 | 3.322516 | 0.02388 | Rpl26 | 4.783673 | 0.044512 | Muc15 | 3.306824 | 0.025805 |
| Ash2l | 1.92501 | 0.010924 | Stag2 | 3.118525 | 0.004987 | Ranbp6 | 3.355852 | 0.027891 | Bax | 3.677817 | 0.015898 |
| Kdm6b | 2.298966 | 0.034146 | Twist1 | 5.766689 | 0.007902 | Mlf1 | 5.521454 | 0.025057 | Ubtd2 | 3.665633 | 0.020212 |
| Cyp4v3 | 3.195162 | 0.02921 | Rala | 5.635887 | 0.016227 | RGD1564420 | 1.630531 | 0.042861 | Akt2 | 2.542033 | 0.017594 |
| Nr1d2 | 2.682145 | 0.040737 | Egfr | 2.729588 | 0.043412 | Slc16a13 | 1.662644 | 0.01787 | Rundc3b | 2.74858 | 0.045943 |
| Ube2n | 5.303738 | 0.020219 | Srcin1 | 2.366873 | 0.041068 | LOC291276 | 2.751501 | 0.039465 | Tfb1m | 1.74783 | 0.011938 |
| Rora | 2.710567 | 0.04252 | Lrrc14 | 1.93197 | 0.025994 | Qrsl1 | 3.271467 | 0.031808 | LOC257642 | 3.309402 | 2.73E-05 |
| Tipinl1 | 5.59709 | 0.010689 | Tyms | 3.159234 | 0.043376 | T2 | 3.239733 | 0.014106 | MGC116197 | 3.311813 | 0.003293 |
| Acsl3 | 2.265736 | 0.03729 | Tmeff2 | 2.869695 | 0.020994 | Kctd14 | 3.342655 | 0.019522 | Gmps | 2.892817 | 0.03305 |
| Bco2 | 1.559351 | 0.044014 | Rpl6-ps1 | 5.399584 | 1.94E-07 | Asb6 | 2.068649 | 0.006525 | Ppif | 5.395078 | 0.019968 |
| Dnajb13 | 1.577257 | 0.033452 | Calr4 | 3.393038 | 0.005384 | Ak7 | 3.53385 | 0.006513 | Calml3 | 1.660281 | 0.047707 |
| Rnf115 | 3.446139 | 0.019886 | Rab5c | 2.827482 | 0.00844 | Yeats2 | 3.212866 | 0.032847 | Cited1 | 3.439263 | 0.003252 |
| Cdk10 | 1.854223 | 0.017654 | Grk6 | 3.551344 | 0.023087 | Gpr25 | 3.055441 | 0.04353 | Angptl4 | 2.619404 | 0.030321 |
| Cystm1 | 2.327625 | 0.038706 | Lamtor2 | 2.903698 | 0.022833 | Acer3 | 3.223464 | 0.049518 | Tns4 | 3.347088 | 0.03011 |
| Mogat1 | 2.497832 | 0.000457 | Rps18l1 | 2.732669 | 0.011871 | **Down-regulated genes** | **Log FC** | ***P*** | Ano3 | 2.771007 | 0.001325 |
| Ifit3 | 3.420361 | 0.026742 | Fgfr1op2 | 3.254585 | 0.023168 | Zfp347 | -2.64464 | 0.047271 | Ubd | 2.67832 | 0.041737 |
| Ccl12 | 3.654789 | 0.012998 | Lhcgr | 3.966575 | 0.001044 | Blmh | -5.00694 | 0.046978 | Slfn4 | 3.32758 | 0.018662 |
| Rnf166 | 1.860668 | 0.035142 | Ppp6c | 2.662965 | 0.000811 | Foxs1 | -2.29304 | 0.005 | LOC257642 | 2.064525 | 0.004089 |
| Prob1 | 3.39071 | 0.028365 | Timm21 | 5.110884 | 0.045017 | Appbp2 | -2.62295 | 0.016907 | Brms1 | 1.826493 | 0.015516 |
| LOC498933 | 2.812192 | 0.005159 | Prr15 | 3.596026 | 0.033001 | Parp2 | -5.05536 | 5.62E-08 | Cenpe | 3.780905 | 0.002834 |
| Hsf1 | 1.606331 | 0.037167 | Snrpb | 2.176356 | 0.026159 | Cnnm1 | -3.61836 | 0.03311 | Fbp1 | 5.574166 | 0.031285 |
| Nfkb1 | 2.423185 | 0.036305 | Upf3b | 1.615621 | 0.048707 | Arrb2 | -3.15636 | 0.016701 | Rgl3 | 5.245733 | 0.031906 |
| Zhx2 | 1.647838 | 0.034658 | Epc2 | 3.58049 | 0.043765 | Wdr4 | -2.11529 | 0.024257 | Wfdc2 | 3.119798 | 0.046189 |
| Noc4l | 2.10807 | 0.005453 | Atp6v0d1 | 5.244287 | 0.029885 | Uqcrb | -3.73612 | 0.003444 | Arhgap26 | 2.143908 | 0.023159 |
| Nags | 1.835013 | 0.041215 | Gpx4 | 2.253788 | 0.017058 | LOC100361655 | -2.47762 | 0.005402 | Gsr | 2.495229 | 0.033213 |
| Camsap2 | 1.830839 | 0.020063 | Atg5 | 3.664346 | 0.016075 | Pfdn1 | -2.35195 | 0.022393 | Suds3 | 4.186049 | 0.00359 |
| Fgfbp3 | 3.942954 | 0.017964 | Gapvd1 | 3.436264 | 0.018279 | Lage3 | -5.12554 | 0.020526 | Sympk | 2.702657 | 0.046159 |
| Igtp | 2.943279 | 0.034706 | Prx | 3.619318 | 0.031275 | Dnase1l3 | -5.71891 | 0.033029 | Cd53 | 3.521216 | 0.030219 |
| Duox1 | 5.166204 | 0.031996 | MGC116197 | 2.70778 | 0.007246 | Fbxo44 | -2.80999 | 0.045492 | Klhdc8a | 2.1131 | 0.010177 |
| Akr1c13 | 2.491961 | 0.000437 | Hnrnpa1 | 2.224 | 0.033637 | N6amt1 | -2.19101 | 0.040953 | Fam131a | 4.175812 | 1.27E-05 |
| LOC100302465 | 2.867567 | 0.000162 | Shisa5 | 2.618152 | 0.046558 | Rpl21 | -2.25554 | 0.048523 | Fabp6 | 2.927272 | 0.002087 |
| Msc | 3.533824 | 0.0196 | Cyhr1 | 1.796971 | 0.03203 | Apopt1 | -4.00177 | 0.009744 | **Down-regulated genes** | **Log FC** | ***P*** |
| Elp2 | 5.452 | 0.020223 | Slco3a1 | 3.600344 | 0.023934 | Hspe1 | -1.7342 | 0.025481 | Trpm7 | -2.84828 | 0.023281 |
| Qrsl1 | 3.495967 | 0.021012 | Pacs1 | 2.037677 | 0.03673 | Sdr9c7 | -3.59927 | 0.027677 | Plk4 | -2.45275 | 0.041602 |
| Rgl3 | 3.089403 | 0.04903 | Fam76b | 5.527256 | 0.035784 | Fam114a2 | -3.59548 | 0.019638 | Exosc4 | -2.07186 | 0.01995 |
| Kctd14 | 3.318801 | 0.020144 | Micu2 | 3.445133 | 0.018575 | LOC257642 | -4.66882 | 4.48E-08 | LOC102552055 | -1.91054 | 0.041532 |
| Asb6 | 2.428413 | 0.001439 | Spdya | 2.668878 | 0.026453 | Osbpl6 | -1.76235 | 0.036808 | Vopp1 | -3.50428 | 0.027191 |
| Gpr25 | 4.058453 | 0.00668 | Wdr63 | 2.679628 | 0.015134 | Dars | -2.44201 | 0.029685 | Eci2 | -4.26359 | 2.98E-08 |
| Slc25a10 | 1.947212 | 0.049204 | Myom2 | 2.725802 | 0.016824 | Klhdc4 | -5.08432 | 0.049595 | Kpna1 | -2.11117 | 0.028905 |
| Myzap | 2.379741 | 0.047441 | Slc38a2 | 2.435566 | 0.029391 | Stmn1 | -2.75128 | 0.010198 | Gcnt3 | -1.62428 | 0.018553 |
| Thg1l | 2.921406 | 0.048466 | Gclc | 2.618052 | 0.014073 | Acat1 | -1.82089 | 0.041033 | Chrna2 | -2.58636 | 0.000626 |
| **Down-regulated genes** | **Log FC** | ***P*** | Rbm15 | 2.750553 | 0.046689 | Lrrcc1 | -1.94788 | 0.030594 | Slc35g2 | -2.0388 | 0.043279 |
| Lmna | -2.23632 | 0.019191 | Cep120 | 5.382414 | 0.026446 | Gch1 | -3.53015 | 0.020155 | Rmdn2 | -2.69501 | 0.008221 |
| Tmed4 | -2.26314 | 0.044142 | Psme3 | 5.429639 | 0.041992 | Hn1 | -2.09119 | 0.030949 | Dtx1 | -1.94828 | 0.012426 |
| Ptgdrl | -1.80192 | 0.040503 | Ubash3b | 2.938627 | 0.035119 | Olr1433 | -6.07475 | 0.000861 | Bcl2a1 | -3.4245 | 0.024818 |
| Cacna1d | -1.80355 | 0.030892 | Ino80e | 2.337168 | 0.002424 | Pdhb | -1.83036 | 0.041303 | Myo10 | -1.69574 | 0.026699 |
| Parp2 | -2.40712 | 0.002078 | Rpap3 | 5.564849 | 0.017035 | Fbxl15 | -1.85165 | 0.048341 | Akr1c14 | -1.53976 | 0.049449 |
| Myh11 | -1.52542 | 0.038772 | Arhgap18 | 2.678845 | 0.011393 | Ncam1 | -2.62095 | 0.036664 | Xrn2 | -2.71388 | 0.007608 |
| Tprg1l | -2.72745 | 0.030324 | Sema3c | 2.125565 | 0.011923 | Ostc | -2.97082 | 0.030486 | RT1-S2 | -1.65806 | 0.028509 |
| Kpna1 | -2.54293 | 0.007941 | Erich2 | 2.885031 | 0.037429 | Hs3st5 | -2.39328 | 0.044636 | Nrcam | -8.57903 | 2.17E-14 |
| Rragc | -3.06508 | 0.038877 | Fabp6 | 2.323865 | 0.011822 | Tbk1 | -3.90672 | 0.012147 | Ebf2 | -3.19417 | 0.038219 |
| Klhl14 | -1.82723 | 0.02195 | Suco | 2.401131 | 0.025626 | Phyh | -2.10024 | 0.041942 | Grk4 | -3.89147 | 0.007915 |
| Sft2d2 | -2.26075 | 0.048986 | Gpnmb | 2.09128 | 0.044854 | Fbxo2 | -3.02076 | 0.02287 | Zfp703 | -3.26259 | 0.026055 |
| Ssfa2 | -2.31296 | 0.032181 | **Down-regulated genes** | **Log FC** | ***P*** | AC121204.1 | -3.34033 | 0.013803 | RGD1359449 | -3.05843 | 0.003765 |
| Copb1 | -2.45174 | 0.037899 | Cyp46a1 | -3.35711 | 0.033142 | Ninl | -3.1394 | 0.044844 | Ube3c | -3.7959 | 0.016113 |
| Aen | -2.31075 | 0.019072 | Arhgap35 | -5.12401 | 0.047787 | LOC102557137 | -2.22614 | 0.025292 | Them5 | -5.60473 | 0.02149 |
| Impact | -2.3213 | 0.049151 | G6b | -2.24444 | 0.037963 | Nptx2 | -3.15773 | 0.018282 | Nell1 | -3.78008 | 9.05E-07 |
| Id4 | -4.98293 | 0.035229 | Yipf6 | -5.79891 | 0.025291 | Sdf2l1 | -3.54742 | 0.03697 | Hipk3 | -1.61717 | 0.02814 |
| Azgp1 | -3.11546 | 0.011853 | Rps6ka1 | -2.0698 | 0.034087 | Slc33a1 | -2.49842 | 0.023776 | Gab1 | -5.46805 | 0.028249 |
| Arhgap9 | -2.23282 | 0.030996 | Sema3g | -1.50746 | 0.043477 | Kif1a | -3.13485 | 0.036673 | Gpr151 | -2.7146 | 0.004257 |
| Zc3h11a | -5.13933 | 0.029789 | Lhx3 | -2.07814 | 0.006355 | Ccdc94 | -2.51025 | 0.027989 | Lrrc75b | -1.47938 | 0.038028 |
| Pbx1 | -2.83612 | 0.044693 | Gnl1 | -2.16068 | 0.021191 | Kif24 | -2.67794 | 0.039506 | Tgif1 | -1.90048 | 0.029739 |
| Dnase1l3 | -5.71866 | 0.040052 | LOC103691261 | -6.24025 | 0.008704 | Igsf11 | -1.67641 | 0.038199 | Prkag2 | -4.25811 | 2.8E-07 |
| Smarca5 | -2.92743 | 0.012791 | Apom | -2.01343 | 0.037545 | Gpsm3 | -2.93242 | 0.032011 | Smoc1 | -2.08141 | 0.044852 |
| AABR07024439.1 | -2.87113 | 0.022722 | Fam71f2 | -2.72758 | 0.040841 | Ddx10 | -2.76346 | 0.03015 | Ntn3 | -3.39082 | 0.027745 |
| Psmd1 | -1.95519 | 0.01864 | Kcnk4 | -1.71864 | 0.043166 | LOC257642 | -3.64082 | 2.23E-05 | Camta1 | -2.33212 | 0.02084 |
| Stk39 | -3.27211 | 0.003702 | Prpf19 | -2.62209 | 0.039067 | Slitrk6 | -3.33167 | 0.033308 | Sema3d | -2.40602 | 0.01851 |
| Kif19 | -3.414 | 0.018137 | Igsf8 | -5.32502 | 0.027916 | Hnrnpa3 | -5.35497 | 0.023602 | Ube3a | -2.76283 | 0.001761 |
| Ivd | -5.19453 | 0.042701 | Hnrnph2 | -2.76404 | 0.024913 | Gpr171 | -5.47545 | 0.016991 | Zswim1 | -2.80775 | 0.009004 |
| Apmap | -2.82949 | 0.026572 | Fntb | -1.86423 | 0.027278 | Cabp1 | -3.32361 | 0.047935 | Gjb6 | -5.40194 | 0.02785 |
| Vti1b | -1.46107 | 0.0447 | Ly6e | -2.64272 | 0.036589 | Sos1 | -2.65571 | 0.025802 | Mark1 | -2.52808 | 0.041731 |
| Ptprcap | -2.94484 | 0.008027 | Zfp78 | -3.08608 | 0.041534 | Mcrs1 | -3.47295 | 0.019276 | Aar2 | -3.10823 | 0.039991 |
| Rtn1 | -3.78229 | 0.012023 | Cox5b | -2.90123 | 0.018378 | Tmcc3 | -3.75927 | 0.028001 | Hgd | -3.50652 | 0.037576 |
| Idua | -3.72449 | 0.012946 | Sema3d | -3.00004 | 0.004118 | Nrn1l | -3.3218 | 0.033811 | Fam219b | -2.30644 | 0.045849 |
| Tsga10ip | -2.91196 | 0.03805 | Gjb6 | -3.80697 | 0.011421 | Arsb | -3.38956 | 0.007526 | Akr1c1 | -5.4665 | 0.025741 |
| Hipk3 | -2.0169 | 0.007837 | Dcaf7 | -2.52953 | 0.034489 | Rbfox3 | -1.76585 | 0.04809 | Pfkl | -2.21862 | 0.042804 |
| Tmem8a | -3.52596 | 0.013279 | Rps6kb2 | -3.65081 | 0.021573 | Cdkn2aip | -2.21569 | 0.041213 | Sema6a | -2.28718 | 0.027033 |
| LOC257642 | -2.83116 | 0.000218 | Rpl17 | -2.51271 | 0.023178 | Tmem70 | -5.82287 | 0.007345 | Msmb | -5.22059 | 0.022899 |
| Ldhd | -2.85993 | 0.046225 | Gli1 | -2.23077 | 0.017687 | Rasal1 | -3.47483 | 0.020691 | Clec12b | -5.43017 | 0.017961 |
| Kcnf1 | -2.78126 | 0.035735 | LOC691807 | -2.91899 | 0.022073 | AABR07015078.2 | -2.91438 | 0.00022 | Zfp385c | -2.06497 | 0.043957 |
| Casc4 | -2.81616 | 0.019206 | Ythdf2 | -3.48474 | 0.015947 | Kcnab3 | -2.64645 | 0.046616 | Cpne2 | -3.82453 | 0.002056 |
| Mrps26 | -2.91517 | 0.033319 | Gbp2 | -3.28421 | 0.010392 | Smap1 | -1.73477 | 0.036535 | Perp | -3.2399 | 0.024183 |
| Gyg1 | -3.25686 | 0.042594 | Pja2 | -2.83835 | 0.039567 | Ifrd2 | -1.71854 | 0.027239 | AABR07070085.1 | -2.22216 | 0.003158 |
| Plxnd1 | -3.22542 | 0.023622 | Upk3bl | -2.44136 | 0.02806 | Cdc25a | -2.78114 | 0.043207 | Fscn2 | -1.62735 | 0.033911 |
| Itpka | -2.87262 | 0.034672 | Speg | -2.08093 | 0.039386 | Mtmr1 | -5.1961 | 0.045617 | Hist2h2ab | -3.46411 | 0.020577 |
| Ctbp1 | -2.25197 | 0.044075 | Tmem216 | -1.88379 | 0.045054 | Zfp52 | -2.92985 | 0.039459 | Apobr | -1.45745 | 0.040348 |
| Nans | -3.45461 | 0.01342 | Snx16 | -5.40669 | 0.032908 | Ldb3 | -2.99185 | 0.034306 | Dbn1 | -1.86624 | 0.031635 |
| Ext2 | -2.76038 | 0.042277 | Reep6 | -2.39903 | 0.008681 | Isca1 | -2.88632 | 0.037324 | Hunk | -3.2928 | 0.024072 |
| Cad | -5.40853 | 0.03991 | Angel2 | -2.42282 | 0.030833 | Mapk14 | -2.98303 | 0.020486 | Aldh1a1 | -3.54672 | 0.008899 |
| Fmo1 | -2.24789 | 0.026358 | Oas1h | -2.84748 | 0.026878 | Tspan12 | -3.13051 | 0.043828 | Arfip2 | -3.3447 | 0.023617 |
| Naa35 | -5.18486 | 0.038251 | Znfx1 | -3.213 | 0.032431 | Hps1 | -3.14964 | 0.021322 | Elmsan1 | -2.00388 | 0.041353 |
| Zmynd10 | -3.90858 | 0.00522 | Ift57 | -2.92439 | 0.049754 | Usp11 | -3.53469 | 0.027899 | Dnajb2 | -2.08468 | 0.013409 |
| Aldh2 | -1.52012 | 0.044633 | Colgalt2 | -4.07771 | 0.009277 | Nefh | -2.86899 | 0.040306 | Gpr22 | -1.95492 | 0.046504 |
| Hpca | -2.86001 | 0.02771 | Lmtk3 | -3.11105 | 0.046725 | Tyw1 | -3.04627 | 0.037525 | Fbxw8 | -2.52843 | 0.018062 |
| Rnf150 | -3.29605 | 0.038771 | Mob3b | -5.23805 | 0.041548 | Alas2 | -3.91273 | 0.008414 | RGD1562310 | -3.75343 | 0.010466 |
| Emb | -5.11822 | 0.036859 | RGD1561149 | -2.92084 | 0.011433 | Abcd3 | -2.56522 | 0.020114 | Cyb5rl | -5.59091 | 0.02243 |
| Pdlim1 | -2.69197 | 0.038111 | P2ry14 | -3.29563 | 0.025046 | Ctr9 | -1.68924 | 0.041624 | Cxcl14 | -5.43366 | 0.016165 |
| Krt16 | -3.34372 | 0.04446 | Mybph | -2.5487 | 0.002219 | Timm13 | -3.2056 | 0.030274 | Efemp2 | -2.34949 | 0.032535 |
| Actr1a | -2.18255 | 0.019979 | Mtcp1 | -3.18064 | 0.031668 | Ubac2 | -2.96208 | 0.035656 | Pik3cd | -3.05586 | 0.011166 |
| Clic1 | -2.29322 | 0.016704 | Tnfaip1 | -2.73871 | 0.019114 | Cep192 | -6.02269 | 0.004386 | Tbc1d22b | -2.0078 | 0.031308 |
| Chn1 | -4.78029 | 0.041986 | Esyt3 | -2.73207 | 0.041972 | Vasn | -2.30656 | 0.044561 | Calr3 | -3.92775 | 0.001021 |
| Stmn1 | -3.10138 | 0.005003 | Dolpp1 | -3.27929 | 0.011202 | Grb14 | -2.5341 | 0.038258 | Dear | -1.78788 | 0.012796 |
| Bmp1 | -2.39341 | 0.042309 | Macrod1 | -2.77052 | 0.047612 | Slc6a7 | -2.97025 | 0.045133 | Mybph | -1.54756 | 0.038897 |
| Cript | -2.45752 | 0.045663 | Hsd17b12 | -5.27312 | 0.044109 | Nup93 | -5.2672 | 0.032125 | Trerf1 | -3.33667 | 0.035967 |
| Fzd6 | -2.56677 | 0.045388 | Atrip | -5.4675 | 0.026258 | Dennd3 | -3.47401 | 0.03199 | Ephx1 | -2.41898 | 0.047231 |
| LOC100911110 | -1.89946 | 0.044666 | Yipf3 | -3.62829 | 0.009518 | Tgfb3 | -2.70764 | 0.032495 | Nrgn | -3.74537 | 0.011111 |
| Alcam | -1.8005 | 0.043565 | Mzf1 | -2.79478 | 0.033681 | Syngr2 | -1.73343 | 0.025599 | Rnf168 | -5.09801 | 0.039899 |
| RGD1359290 | -2.38517 | 0.04978 | Abi3 | -2.64622 | 0.03404 | Iars | -1.65854 | 0.031952 | Fastkd1 | -2.30415 | 0.027455 |
| Cpne7 | -2.79278 | 0.048548 | St6galnac4 | -3.38842 | 0.029232 | Lhcgr | -2.60987 | 0.025953 | Il1rn | -2.99064 | 0.042526 |
| Ifi47 | -2.48516 | 0.022114 | Wdr88 | -5.63804 | 0.020808 | Lgmn | -1.94019 | 0.037391 | Pja1 | -1.99865 | 0.048672 |
| Jade2 | -2.96539 | 0.049299 | Mmp20 | -2.8232 | 0.004114 | Mknk2 | -5.41037 | 0.01759 | Aak1 | -2.64659 | 0.03616 |
| St6gal1 | -3.57562 | 0.003777 | Rara | -2.80438 | 0.038068 | Mtmr2 | -2.7194 | 0.043339 | Hao2 | -1.73023 | 0.020587 |
| Bptf | -2.33708 | 0.035094 | Apol9a | -3.57751 | 0.019383 | Aldh5a1 | -2.4024 | 0.029425 | Lcn6 | -2.19318 | 0.026154 |
| Krt19 | -2.5753 | 0.016822 | Mrps18c | -3.39837 | 0.00927 | Kalrn | -2.00226 | 0.019388 | Senp17 | -2.44342 | 0.018025 |
| Cwc22 | -2.2954 | 0.034505 | Cgnl1 | -2.2591 | 0.020752 | Stard4 | -5.23619 | 0.043902 | AC121204.4 | -2.45114 | 0.048648 |
| Hn1 | -2.5079 | 0.011991 | Lrp8 | -2.18717 | 0.030546 | Crabp2 | -2.90493 | 0.015171 | Tanc1 | -3.13528 | 0.026888 |
| LOC103689986 | -1.96682 | 0.010427 | Tmprss2 | -5.97668 | 1.11E-11 | Tfb1m | -2.95045 | 7.27E-05 | Ttc22 | -3.99715 | 4.72E-06 |
| Rpl13a | -1.53108 | 0.045201 | Trmt10a | -3.15122 | 0.036629 | LOC257642 | -3.94808 | 1.78E-06 | Scd | -2.79308 | 0.017521 |
| Phax | -3.77 | 0.009698 | Acan | -1.46106 | 0.039509 | Ppif | -5.01889 | 0.035939 | Parp9 | -1.85467 | 0.027824 |
| Rap2a | -5.4895 | 0.020488 | Lmf2 | -2.01437 | 0.032816 | AABR07036855.1 | -3.38051 | 0.044162 | Slc7a8 | -3.74963 | 0.001532 |
| Cnpy2 | -2.47819 | 0.017046 | Znf48 | -3.53674 | 0.033171 | Calml3 | -1.70131 | 0.044413 | AABR07018272.1 | -2.84059 | 0.037119 |
| Plet1 | -1.7839 | 0.014825 | Sbno1 | -3.8263 | 0.004082 | Hes6 | -3.1088 | 0.02002 | Sept12 | -2.21673 | 0.011051 |
| Enkur | -5.25472 | 0.015513 | Kdm6b | -2.16571 | 0.048053 | Rad51d | -3.96029 | 0.015542 | Agfg2 | -3.1748 | 0.009028 |
| Fam107a | -2.13878 | 0.041991 | Urb1 | -1.74303 | 0.045689 | Matk | -2.65405 | 0.033683 | Atrip | -5.51649 | 0.021045 |
| Pcsk6 | -3.0348 | 0.045951 | Topaz1 | -3.61651 | 0.029962 | LOC257642 | -4.53663 | 2.26E-08 | Hbb | -5.26454 | 0.029487 |
| Ccny | -1.74709 | 0.023452 | Upk3b | -1.81251 | 0.028854 | Rad54b | -4.97511 | 0.034355 | Akr1c19 | -1.71604 | 0.015558 |
| Pdhb | -2.1279 | 0.019865 | Slc20a1 | -2.80232 | 0.027637 | Cenpe | -2.89098 | 0.023065 | Gcdh | -3.52408 | 0.010453 |
| Ostc | -3.23537 | 0.022135 | Prob1 | -3.10429 | 0.048469 | Papola | -2.34958 | 0.020757 | Psmc4 | -4.07326 | 0.00256 |
| Rbm7 | -5.41379 | 0.014395 | RGD1309106 | -2.30384 | 0.01246 | Fam13c | -3.52326 | 0.031291 | Dbndd1 | -5.06402 | 0.0463 |
| Celsr2 | -2.07175 | 0.038145 | Arhgef7 | -3.17638 | 0.045416 | Gsr | -3.18874 | 0.006405 | Ubl3 | -3.01701 | 0.016168 |
| Tbc1d22b | -2.84065 | 0.005477 | Tmem179 | -2.44108 | 0.008044 | Ino80e | -1.69798 | 0.026347 | Adamts5 | -1.81728 | 0.040661 |
| Ndufa9 | -2.86631 | 0.046704 | Map3k5 | -4.97613 | 0.042291 | Klhdc8a | -2.25078 | 0.00701 | Ka11 | -3.85164 | 0.003449 |
| Arf1 | -2.06475 | 0.035063 | Bok | -3.07073 | 0.040843 | Fam131a | -3.15277 | 0.001036 | Sntb2 | -3.55208 | 0.028851 |
| Sult2b1 | -1.81191 | 0.012358 | Stxbp5 | -2.75672 | 0.021837 | Gpnmb | -2.83398 | 0.007356 | Dpy19l1 | -5.25973 | 0.033296 |
| Ndufa5 | -2.80436 | 0.038605 | Sncg | -3.0622 | 0.008991 |  |  |  | Paxbp1 | -2.92196 | 0.041267 |
| Ofd1 | -3.14127 | 0.012359 | Ptp4a2 | -3.18715 | 0.006827 |  |  |  | Bcl6b | -1.90082 | 0.007495 |
| Kcna2 | -3.0124 | 0.039462 | Ngly1 | -2.55865 | 0.037267 |  |  |  | Degs1 | -2.7023 | 0.006134 |
| Ifih1 | -2.20069 | 0.047261 | Six5 | -3.31181 | 0.031171 |  |  |  | Igfbp3 | -5.47379 | 0.034779 |
| Las1l | -1.94724 | 0.039274 | Ybey | -1.45748 | 0.045275 |  |  |  | RGD1308775 | -3.5264 | 0.019552 |
| Kif1a | -3.68062 | 0.020408 | Fgfbp3 | -5.66376 | 0.042549 |  |  |  | Etv6 | -2.23419 | 0.030953 |
| Lmo3 | -3.4527 | 0.00752 | Gpr107 | -3.02794 | 0.044232 |  |  |  | Kcnj8 | -5.29622 | 0.044857 |
| Ndufaf4 | -5.48353 | 0.037883 | Eci1 | -2.80694 | 0.02623 |  |  |  | Strn3 | -3.09716 | 0.031902 |
| Susd2 | -4.88387 | 0.043057 | Tnnt2 | -2.02545 | 0.034716 |  |  |  | Tor3a | -3.37963 | 0.023761 |
| Ahctf1 | -2.7256 | 0.034182 | Sumf1 | -5.09535 | 0.043093 |  |  |  | Abl2 | -2.24693 | 0.024543 |
| Tnrc6b | -2.32767 | 0.043506 | Ubd | -3.4659 | 0.008079 |  |  |  | Ppp3cb | -3.4757 | 0.015926 |
| Yif1b | -5.40579 | 0.037303 | Hnrnpa3 | -2.03884 | 0.015645 |  |  |  | Scx | -2.07188 | 0.018374 |
| Glb1 | -3.24509 | 0.023867 | Lypd1 | -1.58592 | 0.023341 |  |  |  | Lrmp | -3.13374 | 0.01376 |
| Ptbp1 | -5.22158 | 0.011102 | Phyhipl | -2.33202 | 0.023225 |  |  |  | Smagp | -2.77918 | 0.012272 |
| Lcn6 | -2.84187 | 0.017364 | Arhgap24 | -5.5858 | 0.025362 |  |  |  | Mbtps2 | -2.51683 | 0.032006 |
| Man2b1 | -1.96401 | 0.049564 | Irf3 | -2.71614 | 0.038435 |  |  |  | Vdac3 | -2.65175 | 0.039115 |
| Rbx1 | -2.64306 | 0.039929 | Abcc2 | -2.98302 | 0.048102 |  |  |  | Tm2d2 | -5.1656 | 0.041571 |
| Fcho1 | -2.46164 | 0.017548 | Rarg | -3.3617 | 0.002792 |  |  |  | Map4k2 | -3.28626 | 0.025245 |
| Gpt | -2.92304 | 0.014427 | Mx2 | -2.10434 | 0.009464 |  |  |  | Acan | -1.58663 | 0.026188 |
| Emc10 | -2.74112 | 0.046579 | Shmt2 | -3.31292 | 0.024057 |  |  |  | Itsn1 | -3.4729 | 0.005857 |
| Slf2 | -2.86033 | 0.019763 |  |  |  |  |  |  | Sbno1 | -2.42648 | 0.026691 |
| AC115322.1 | -3.31939 | 0.022713 |  |  |  |  |  |  | Itga3 | -1.64784 | 0.042233 |
| LOC257642 | -2.05867 | 0.00817 |  |  |  |  |  |  | Rbm28 | -2.56301 | 0.049717 |
| Slitrk6 | -5.13968 | 0.046819 |  |  |  |  |  |  | Fam13a | -2.11617 | 0.04772 |
| Wasf2 | -3.16231 | 0.021234 |  |  |  |  |  |  | Pde7a | -2.04344 | 0.042207 |
| Brpf1 | -2.27495 | 0.025403 |  |  |  |  |  |  | Snx19 | -3.25863 | 0.049075 |
| Atxn10 | -5.43858 | 0.014187 |  |  |  |  |  |  | Pik3c2b | -3.2939 | 0.021028 |
| Hbb | -3.40006 | 0.020191 |  |  |  |  |  |  | Hspa1l | -3.15968 | 0.015683 |
| Sarnp | -2.40792 | 0.016735 |  |  |  |  |  |  | Dnajb13 | -1.82811 | 0.015299 |
| Kdm4b | -3.27094 | 0.036678 |  |  |  |  |  |  | Htra4 | -1.7255 | 0.038078 |
| Ap3b2 | -2.52734 | 0.048064 |  |  |  |  |  |  | Siglec15 | -2.58114 | 0.011544 |
| E2f3 | -3.30542 | 0.041693 |  |  |  |  |  |  | Prpf18 | -2.55505 | 0.017432 |
| Psmc4 | -3.92745 | 0.00361 |  |  |  |  |  |  | Mogat1 | -2.5291 | 0.000308 |
| Mrpl39 | -2.15291 | 0.027046 |  |  |  |  |  |  | AABR07012635.1 | -2.44262 | 0.000928 |
| Tnks1bp1 | -2.3996 | 0.044191 |  |  |  |  |  |  | Adamts12 | -3.3799 | 2.65E-06 |
| Lamb2 | -1.76298 | 0.038907 |  |  |  |  |  |  | Rpl6-ps1 | -5.35673 | 2.41E-07 |
| Tmcc3 | -3.54489 | 0.037593 |  |  |  |  |  |  | Atp6v1b1 | -2.35689 | 0.03489 |
| Dguok | -3.06764 | 0.006989 |  |  |  |  |  |  | Mical2 | -2.87872 | 0.024968 |
| Ppip5k2 | -2.83661 | 0.034748 |  |  |  |  |  |  | AABR07035203.1 | -2.19644 | 0.026985 |
| Dkk3 | -3.43679 | 0.001197 |  |  |  |  |  |  | Secisbp2 | -3.43414 | 0.020058 |
| Ap3s1 | -2.99631 | 0.028683 |  |  |  |  |  |  | Rnf166 | -2.23074 | 0.013574 |
| Smim19 | -3.04468 | 0.023042 |  |  |  |  |  |  | Prob1 | -3.29943 | 0.034598 |
| Lsg1 | -3.65565 | 0.023234 |  |  |  |  |  |  | Epas1 | -2.38586 | 0.02828 |
| Slc25a11 | -5.87802 | 0.007409 |  |  |  |  |  |  | Cd55 | -1.94185 | 0.043364 |
| Fads2 | -2.44533 | 0.017557 |  |  |  |  |  |  | Adam30 | -2.36695 | 0.039099 |
| Rftn1 | -3.40109 | 0.022039 |  |  |  |  |  |  | C1qtnf1 | -3.48869 | 0.001204 |
| Zfp57 | -2.26371 | 0.010654 |  |  |  |  |  |  | Dhcr24 | -2.44802 | 0.005551 |
| Iqcd | -5.01877 | 0.032963 |  |  |  |  |  |  | Rps10 | -5.04507 | 0.048076 |
| AABR07015078.2 | -1.80089 | 0.015508 |  |  |  |  |  |  | Zhx2 | -1.82677 | 0.020265 |
| St3gal5 | -2.47056 | 0.026271 |  |  |  |  |  |  | Dfnb31 | -2.39655 | 0.017417 |
| Uqcrh | -2.2472 | 0.013667 |  |  |  |  |  |  | Glmn | -1.54867 | 0.045304 |
| Nap1l1 | -2.36988 | 0.012808 |  |  |  |  |  |  | RGD1565283 | -1.85626 | 0.041272 |
| Smap1 | -1.76553 | 0.033787 |  |  |  |  |  |  | Camsap2 | -2.04868 | 0.016045 |
| Ifrd2 | -4.08667 | 8.33E-06 |  |  |  |  |  |  | Cyb5r1 | -1.6323 | 0.030849 |
| Rsl24d1 | -3.25022 | 0.038396 |  |  |  |  |  |  | Dusp1 | -2.89204 | 0.033909 |
| Gramd1a | -2.20125 | 0.027833 |  |  |  |  |  |  | Dhrs7 | -5.7052 | 0.012226 |
| Nrxn2 | -1.66124 | 0.039209 |  |  |  |  |  |  | Lyplal1 | -3.1964 | 0.018595 |
| Mtmr1 | -3.34924 | 0.042205 |  |  |  |  |  |  | AABR07061254.1 | -2.79614 | 0.014079 |
| Txndc16 | -5.65158 | 0.020094 |  |  |  |  |  |  | Cox7c | -3.56064 | 0.005101 |
| Izumo1 | -2.31907 | 0.023906 |  |  |  |  |  |  | Megf8 | -2.18412 | 0.035865 |
| Sfrp2 | -2.93679 | 0.049408 |  |  |  |  |  |  | LOC100302465 | -3.44191 | 8.43E-06 |
| Fam117a | -3.18091 | 0.036874 |  |  |  |  |  |  | Abcc4 | -1.8468 | 0.044728 |
| Dapp1 | -2.80853 | 0.042614 |  |  |  |  |  |  | Snx15 | -3.6104 | 0.004598 |
| RGD1559904 | -5.9342 | 0.003587 |  |  |  |  |  |  | Slc16a13 | -2.28726 | 0.001066 |
| Tmem151b | -1.95069 | 0.032389 |  |  |  |  |  |  | Prss53 | -1.74082 | 0.045385 |
| Pxn | -2.35683 | 0.03791 |  |  |  |  |  |  | Aga | -3.38196 | 0.016901 |
| Atp2b1 | -2.47873 | 0.007217 |  |  |  |  |  |  | AC115420.4 | -2.03875 | 0.04698 |
| Col5a2 | -3.09481 | 0.001537 |  |  |  |  |  |  | RGD1566368 | -1.93163 | 0.048719 |
| Wdfy2 | -2.35543 | 0.025953 |  |  |  |  |  |  | Rhov | -3.64633 | 0.005535 |
| Bgn | -1.89218 | 0.033405 |  |  |  |  |  |  | Mrps18b | -2.35355 | 0.002355 |
| Spata6 | -5.20407 | 0.0376 |  |  |  |  |  |  | AC115371.1 | -2.03567 | 0.00488 |
| Fbxo11 | -2.45496 | 0.032506 |  |  |  |  |  |  | Fndc3b | -3.32678 | 0.006908 |
| Pkig | -2.00775 | 0.030586 |  |  |  |  |  |  | Sars2 | -2.01806 | 0.002893 |
| Golph3 | -2.04021 | 0.019618 |  |  |  |  |  |  | Asb6 | -1.5002 | 0.039668 |
| Cyc1 | -2.49818 | 0.011278 |  |  |  |  |  |  | LOC690126 | -3.26458 | 0.004387 |
| Ogt | -3.37241 | 0.001803 |  |  |  |  |  |  | Nav3 | -3.65445 | 0.027128 |
| Snrnp35 | -1.90557 | 0.028018 |  |  |  |  |  |  | Mfge8 | -1.73069 | 0.018059 |
| Hps1 | -3.71935 | 0.010932 |  |  |  |  |  |  | Snn | -2.25289 | 0.041102 |
| Ddx47 | -1.98627 | 0.047599 |  |  |  |  |  |  |  |  |  |
| Stag2 | -2.60421 | 0.02009 |  |  |  |  |  |  |  |  |  |
| Alas2 | -2.93942 | 0.03418 |  |  |  |  |  |  |  |  |  |
| Rala | -5.19775 | 0.032758 |  |  |  |  |  |  |  |  |  |
| Gcc2 | -2.84723 | 0.031465 |  |  |  |  |  |  |  |  |  |
| Gltp | -2.90271 | 0.033466 |  |  |  |  |  |  |  |  |  |
| Adcy6 | -3.24144 | 0.022295 |  |  |  |  |  |  |  |  |  |
| Rnf144b | -5.11526 | 0.027293 |  |  |  |  |  |  |  |  |  |
| Faap24 | -1.81615 | 0.020556 |  |  |  |  |  |  |  |  |  |
| Asb15 | -3.15571 | 0.026882 |  |  |  |  |  |  |  |  |  |
| Htr1b | -2.70744 | 0.040198 |  |  |  |  |  |  |  |  |  |
| Tmeff2 | -3.15332 | 0.010607 |  |  |  |  |  |  |  |  |  |
| Rpl6-ps1 | -6.41139 | 1.33E-09 |  |  |  |  |  |  |  |  |  |
| Rab5c | -2.34548 | 0.029741 |  |  |  |  |  |  |  |  |  |
| Crebrf | -5.26142 | 0.042134 |  |  |  |  |  |  |  |  |  |
| Tubb4b | -1.90332 | 0.036874 |  |  |  |  |  |  |  |  |  |
| Dennd3 | -3.26079 | 0.0442 |  |  |  |  |  |  |  |  |  |
| Tshb | -3.35334 | 0.034532 |  |  |  |  |  |  |  |  |  |
| Fzd1 | -5.17428 | 0.039981 |  |  |  |  |  |  |  |  |  |
| Sh3bgr | -2.47971 | 0.014644 |  |  |  |  |  |  |  |  |  |
| Gnpat | -3.19732 | 0.005794 |  |  |  |  |  |  |  |  |  |
| Cd55 | -2.97818 | 0.004885 |  |  |  |  |  |  |  |  |  |
| Srsf5 | -1.52543 | 0.047252 |  |  |  |  |  |  |  |  |  |
| Psma6 | -2.45254 | 0.049814 |  |  |  |  |  |  |  |  |  |
| Rps18l1 | -2.39701 | 0.028148 |  |  |  |  |  |  |  |  |  |
| Fgfr1op2 | -3.32931 | 0.019621 |  |  |  |  |  |  |  |  |  |
| Lhcgr | -2.91762 | 0.017225 |  |  |  |  |  |  |  |  |  |
| Slc25a20 | -3.75906 | 0.009491 |  |  |  |  |  |  |  |  |  |
| Dlg5 | -3.60703 | 0.01347 |  |  |  |  |  |  |  |  |  |
| Etl4 | -2.48179 | 0.023489 |  |  |  |  |  |  |  |  |  |
| Rps10 | -3.54429 | 0.018194 |  |  |  |  |  |  |  |  |  |
| Rnf14 | -3.39584 | 0.009098 |  |  |  |  |  |  |  |  |  |
| Fam24a | -2.73894 | 0.017304 |  |  |  |  |  |  |  |  |  |
| Crip2 | -3.45606 | 0.009851 |  |  |  |  |  |  |  |  |  |
| Tomm20 | -3.06844 | 0.008874 |  |  |  |  |  |  |  |  |  |
| Cstf2t | -3.52392 | 0.015681 |  |  |  |  |  |  |  |  |  |
| Tapt1 | -3.86725 | 0.008942 |  |  |  |  |  |  |  |  |  |
| Abhd17a | -3.27593 | 0.009256 |  |  |  |  |  |  |  |  |  |
| S1pr1 | -5.25205 | 0.037521 |  |  |  |  |  |  |  |  |  |
| Gtf3c4 | -3.29555 | 0.040175 |  |  |  |  |  |  |  |  |  |
| Afg3l1 | -3.80165 | 0.010091 |  |  |  |  |  |  |  |  |  |
| Skil | -2.80046 | 0.028106 |  |  |  |  |  |  |  |  |  |
| Grhl2 | -5.61287 | 0.017738 |  |  |  |  |  |  |  |  |  |
| Ppp6c | -2.30196 | 0.003936 |  |  |  |  |  |  |  |  |  |
| Plekha5 | -2.44378 | 0.046011 |  |  |  |  |  |  |  |  |  |
| Afmid | -3.58949 | 0.005089 |  |  |  |  |  |  |  |  |  |
| Lpar3 | -4.87257 | 0.048466 |  |  |  |  |  |  |  |  |  |
| Cep70 | -3.55581 | 0.028919 |  |  |  |  |  |  |  |  |  |
| Gpx4 | -1.97719 | 0.037398 |  |  |  |  |  |  |  |  |  |
| RGD1310587 | -1.80576 | 0.040766 |  |  |  |  |  |  |  |  |  |
| Vav2 | -3.42866 | 0.033128 |  |  |  |  |  |  |  |  |  |
| S100a16 | -2.52308 | 0.022809 |  |  |  |  |  |  |  |  |  |
| Srsf10 | -2.19253 | 0.029565 |  |  |  |  |  |  |  |  |  |
| LOC257642 | -1.95704 | 0.008321 |  |  |  |  |  |  |  |  |  |
| Rcn3 | -3.70528 | 0.011722 |  |  |  |  |  |  |  |  |  |
| Gapvd1 | -3.43883 | 0.018085 |  |  |  |  |  |  |  |  |  |
| Inha | -1.94864 | 0.015106 |  |  |  |  |  |  |  |  |  |
| Ppp1r15a | -2.88355 | 0.031642 |  |  |  |  |  |  |  |  |  |
| Gosr1 | -3.18357 | 0.033509 |  |  |  |  |  |  |  |  |  |
| Hnrnpa1 | -3.06093 | 0.003089 |  |  |  |  |  |  |  |  |  |
| Copz1 | -2.25791 | 0.04818 |  |  |  |  |  |  |  |  |  |
| Efnb2 | -6.31277 | 0.001338 |  |  |  |  |  |  |  |  |  |
| Gmps | -5.22432 | 0.032218 |  |  |  |  |  |  |  |  |  |
| Nop58 | -2.30987 | 0.007692 |  |  |  |  |  |  |  |  |  |
| Paip2 | -2.09282 | 0.010288 |  |  |  |  |  |  |  |  |  |
| Shisa5 | -2.65432 | 0.043153 |  |  |  |  |  |  |  |  |  |
| Slc1a1 | -1.75477 | 0.02622 |  |  |  |  |  |  |  |  |  |
| Nsun5 | -5.38058 | 0.018308 |  |  |  |  |  |  |  |  |  |
| Cox7c | -2.82976 | 0.026217 |  |  |  |  |  |  |  |  |  |
| Ttf1 | -2.02245 | 0.008716 |  |  |  |  |  |  |  |  |  |
| Chmp2b | -5.18013 | 0.039905 |  |  |  |  |  |  |  |  |  |
| Lrrc16a | -4.9792 | 0.048231 |  |  |  |  |  |  |  |  |  |
| Pdrg1 | -2.21078 | 0.025421 |  |  |  |  |  |  |  |  |  |
| Fgfr2 | -2.24552 | 0.049823 |  |  |  |  |  |  |  |  |  |
| Micu2 | -2.89996 | 0.047865 |  |  |  |  |  |  |  |  |  |
| Ndufb8 | -2.76853 | 0.017237 |  |  |  |  |  |  |  |  |  |
| Wdr63 | -2.44435 | 0.028025 |  |  |  |  |  |  |  |  |  |
| Tnnt2 | -1.73125 | 0.033098 |  |  |  |  |  |  |  |  |  |
| LOC257642 | -2.75311 | 0.000189 |  |  |  |  |  |  |  |  |  |
| Ilf3 | -2.90344 | 0.01256 |  |  |  |  |  |  |  |  |  |
| Ufsp2 | -2.96632 | 0.04995 |  |  |  |  |  |  |  |  |  |
| Xpnpep1 | -4.93114 | 0.037994 |  |  |  |  |  |  |  |  |  |
| Slc38a2 | -2.87812 | 0.009465 |  |  |  |  |  |  |  |  |  |
| Gclc | -2.69548 | 0.011444 |  |  |  |  |  |  |  |  |  |
| Serpine2 | -2.08014 | 0.037972 |  |  |  |  |  |  |  |  |  |
| Osbpl2 | -2.92298 | 0.020512 |  |  |  |  |  |  |  |  |  |
| Setx | -5.26089 | 0.023374 |  |  |  |  |  |  |  |  |  |
| Eri2 | -1.56246 | 0.022811 |  |  |  |  |  |  |  |  |  |
| Ino80e | -2.24406 | 0.003963 |  |  |  |  |  |  |  |  |  |
| Cfap20 | -2.5388 | 0.023932 |  |  |  |  |  |  |  |  |  |
| Clic6 | -5.68515 | 0.00461 |  |  |  |  |  |  |  |  |  |
| Ptpru | -5.59748 | 0.009354 |  |  |  |  |  |  |  |  |  |
| Rnpep | -3.45642 | 0.015733 |  |  |  |  |  |  |  |  |  |
| Rbm27 | -5.0486 | 0.043932 |  |  |  |  |  |  |  |  |  |
| Farp1 | -2.52754 | 0.040864 |  |  |  |  |  |  |  |  |  |
| Acadm | -2.30715 | 0.004999 |  |  |  |  |  |  |  |  |  |
| Gabarapl1 | -1.7828 | 0.038435 |  |  |  |  |  |  |  |  |  |
| Snrnp48 | -5.78629 | 0.011212 |  |  |  |  |  |  |  |  |  |
| Sema3c | -2.57883 | 0.002573 |  |  |  |  |  |  |  |  |  |
| Fam131a | -1.91115 | 0.030373 |  |  |  |  |  |  |  |  |  |
| Nsf | -3.40173 | 0.037711 |  |  |  |  |  |  |  |  |  |
| RGD1563956 | -3.95403 | 0.005203 |  |  |  |  |  |  |  |  |  |
| Suco | -2.63611 | 0.014081 |  |  |  |  |  |  |  |  |  |
| Gpnmb | -2.47397 | 0.017691 |  |  |  |  |  |  |  |  |  |
